# Can You Play with Fire and Not Hurt Yourself? A Comparative Study in Figurative Language Comprehension between Individuals with and without Autism Spectrum Disorder

**DOI:** 10.1371/journal.pone.0168571

**Published:** 2016-12-30

**Authors:** Sobh Chahboun, Valentin Vulchanov, David Saldaña, Hendrik Eshuis, Mila Vulchanova

**Affiliations:** 1 Language Acquisition and Language Processing Lab, Department of Language and Literature, Norwegian University of Science and Technology, Trondheim, Norway; 2 Language, Cognition and Individual Differences Lab, Department of Educational Psychology, Faculty of Psychology, University of Seville, Seville, Spain; University of Akron, UNITED STATES

## Abstract

Individuals with High functioning autism (HFA) are distinguished by relative preservation of linguistic and cognitive skills. However, problems with pragmatic language skills have been consistently reported across the autistic spectrum, even when structural language is intact. Our main goal was to investigate how highly verbal individuals with autism process figurative language and whether manipulation of the stimuli presentation modality had an impact on the processing. We were interested in the extent to which visual context, e.g., an image corresponding either to the literal meaning or the figurative meaning of the expression may facilitate responses to such expressions. Participants with HFA and their typically developing peers (matched on intelligence and language level) completed a cross-modal sentence-picture matching task for figurative expressions and their target figurative meaning represented in images. We expected that the individuals with autism would have difficulties in appreciating the non-literal nature of idioms and metaphors, despite intact structural language skills. Analyses of accuracy and reaction times showed clearly that the participants with autism performed at a lower level than their typically developing peers. Moreover, the modality in which the stimuli were presented was an important variable in task performance for the more transparent expressions. The individuals with autism displayed higher error rates and greater reaction latencies in the auditory modality compared to the visual stimulus presentation modality, implying more difficulty. Performance differed depending on type of expression. Participants had more difficulty understanding the culturally-based expressions, but not expressions grounded in human experience (biological idioms). This research highlights the importance of stimulus presentation modality and that this can lead to differences in figurative language comprehension between typically and atypically developing individuals. The current study also contributes to current debates on the role of structural language in figurative language comprehension in autism.

## Introduction

Figurative language is a pervasive phenomenon in daily communication, and is reflected in a range of expressions, such as metaphors, idioms, clichés, irony, jokes. Such expressions vary in structure and degrees of transparency and familiarity, and can fluctuate from a single word to a complex sentence. A common feature that unites the categories of figurative language is the fact that reference in such expressions is indirect. More importantly, the interpretation of figurative expressions is non-compositional (non-literal). This deviation from compositional on-line computation and the heterogeneity of figurative uses makes figurative language more demanding in terms of processing [1]. In language development, the ability to interpret figurative language develops gradually, and comprehension of such expressions can be especially demanding in deficit populations where language is affected.

Individuals with High Functioning Autism (HFA) are distinguished by relative preservation of language and cognitive skills. Intelligence in this group is at normal or above average levels, and quite often such individuals demonstrate specific advantages in certain cognitive areas. The term high functioning captures the level of adequate cognitive functioning in that group, while often, when referring to the level of language competence (i.e., intact structural language), they are described as highly verbal. However, problems with pragmatic language have been consistently reported across the autistic spectrum, even when structural language is intact [2–6]. In addition, some studies have found evidence of weaknesses in the figurative and idiomatic language domains, contrasting with clear strengths in areas of grammar [4, 6].

Several behavioral and imaging studies have shown that there are many issues to take into account regarding the interpretation of figurative language. It depends on the type of expression, its degree of compositionality/transparency, its linguistic structure, its source domain of knowledge, and not least, its novelty/conventionality. Thus, the main question is how these factors impact on the comprehension of the categories of interest (idioms, metaphors, irony), and whether the dissociations between structural language skills and figurative language competence reported in highly verbal individuals with autism are still maintained with a careful choice of experimental paradigm.

The current study is designed to answer these questions by focusing on figurative language processing in highly verbal individuals with autism. The main goal was to investigate whether individuals with autism and preserved language competence understand idiomatic expressions and novel metaphors at the same level as carefully matched controls, and, specifically, whether in this process they benefit from visually presented context and can integrate information from multiple sources (e.g. visual modality and oral language presented simultaneously).

### Idiomatic expressions and their processing

The mechanisms underlying the processing of figurative language have been widely debated. Idiomatic expressions are one of the most common and popular forms of figurative language used in daily interaction. Idioms are multi-word expressions whose interpretation is non-literal, meaning it cannot be derived by using regular compositional processes. As such, they require the language user to go beyond what is stated literally in order to infer the communicative intent of the speaker [7]. In addition, idioms have attracted attention both on theoretical and empirical grounds. The reason is that they cannot be placed immediately within a grammar-lexicon dichotomy of language. On the one hand, it can be assumed that they belong in, and are retrieved from, the mental lexicon, following the assumption that they need to be acquired and stored in the same way as lexical items. On the other, their (complex) structure suggests that they may be processed like structures generated by grammar [8–9].

Idiom processing has been addressed extensively in recent research, but mainly, two different types of theoretical approaches have emerged regarding the processing and comprehension of idioms [10]. Representative of the first type of approach is the Lexical Representation Hypothesis. Originally proposed by Swinney & Cutler [11] (see also Bobrow & Bell [12] and Gibbs [13]), this hypothesis claims that idioms are stored as lexical items, and understanding an idiom involves two parallel processes, a retrieval process, and a literal compositional computation process based on decomposing every element separately [8–10]. Further, it is assumed that the retrieval process is faster, while the compositional process demands longer time.

In a further development, Hamblin & Gibbs [14] suggest that idioms are essentially decomposable. For these authors, idiomatic interpretation rests on identifying the individual constituents, mainly because of the decomposability of the idiomatic expressions. Along similar lines, some authors have claimed that the processing and understanding of idioms cannot be reduced to lexical access or lexical retrieval only, and an additional interpretation process might be necessary [15–17]. Thus, Gibbs [16] suggests that idioms invoke complex meanings which are motivated by independently existing conceptual metaphors which are part of everyday thought.

The second type of approach, labelled the Configuration Hypothesis, aims to capture the dynamic process involved in idiom interpretation. The authors in favour of this position argue that idioms are represented in a concrete and distributed way and are processed as complex expressions [15]. This latter approach can be described as a “compositional” approach whereby each constituent is assumed to contribute semantically to the idiom interpretation.

In a comprehensive review of approaches to idioms, Titone & Connine [7] suggest that either view fails to account adequately for findings in research or capture the nature of idioms. These authors thus suggest a “hybrid” model, which acknowledges both the arbitrary “word” nature and the compositional aspect of idioms. Their model takes into account an important parameter on which idioms vary: degree of decomposability. A similar view, albeit from a slightly different perspective, is defended in Sanford [18] who describes idioms as the intersection of conceptual and syntactic schemas. Another factor which has been shown to impact on idiom processing is the mode of presentation. Tabossi, Fanari & Wolf [19] found that idiom identification differs depending on whether the expression was presented auditorily or visually (as text). This clearly implies that the different modalities in which the idioms are presented, demand different forms of processing.

Another related figurative and formulaic language category are proverbs. Proverbs are fixed sentential expressions which invoke well-received wisdom, social or moral norms [20–21]. They reflect themes from everyday life and often refer to general principles of reasonable human behaviour and action [21–22], and, as such, may seem to express self-evident truths. However, like idioms, proverbs display a wide range of interpretations depending on the type of mechanism involved: they can be metaphorical, as *You can’t judge a book by its cover*, or based on personification, hyperbole or a paradox (*No news is good news*). Unlike idioms, however, proverbs are typically based on some kind of cause-effect reasoning (*Once bitten*, *twice shy*) or reasoning by analogy, but may also reflect a certain type of cultural practice (*Never look a gift horse in the mouth*). Although proverbs may have prototypical figurative meanings, they are very much vital due to the necessity to draw precise inferences in each specific context of encounter [21]. Thus, the correct interpretation of a proverb requires the ability to navigate between their concrete, literal meaning and their figurative meaning by establishing a metaphorical connection between these two interpretations. These properties of proverbs make them an interesting category to study, especially in comparison to idioms. In the current study we refer to this type of figurative expression as “instructive expressions” reflecting their communicative role in language use.

Unlike idioms and proverbs, metaphors are a category which is often less conventionalised and more transparent. It is often assumed that metaphors are a device which maps concepts from two distinct domains, which are not otherwise conceptually linked to each other [23]. Like idioms, however, metaphors vary in degree of novelty/conventionality which affects the way they are processed. In the current study we use novel metaphors as a category which does not depend on stored vocabulary knowledge, but rather invites active processing based on analogy. Thus, the processing of novel metaphors can act as a base-line for the processing of novel expressions, in contrast to all other idiomatic categories included in the study.

### Figurative language in High Functioning Autism (HFA)

Despite the long-standing interest in idioms, their nature and processing in typical populations, research on idiom comprehension in atypical populations overall, and in autism, in particular, has been surprisingly scanty.

Mashal & Kasirer [24] conducted a study where individuals with HFA, children with learning disorder and typically developing controls were compared. The authors used 11 subtests, ranging from interpretation of figurative expressions, including visual metaphors, idioms, conventional metaphors, novel metaphors, to comprehension of homophones and semantic tests (synonymy, similarity). Their results showed that children with learning deficit scored significantly lower than typically developing peers on comprehension of conventional metaphors and idioms, but no significant differences were observed between the two groups on comprehension of visual and novel metaphors. Furthermore, both deficit groups displayed a dissociation between the visual modality and the auditory modality, supporting the assumption that modality is a salient factor in figurative language processing. In contrast, the control group demonstrated an association between idioms and conventional metaphors. This result supports the idea that idioms and conventional metaphors share a common ground, in that both are relatively frozen conventionalised expressions and most likely rest on similar mechanisms for their processing. This is consistent with findings in a recent study of metaphor processing in highly verbal individuals with autism [25].

Vogindroukas & Zikopoulou [26] conducted a study aimed at the developmental trajectory of idiom comprehension in Asperger’s syndrome (AS) and HFA. The authors compared children with HFA/AS to their typically developing peers and to control adults. They found that children with AS/ HFA performed more poorly on the idiom task compared to the other two groups, while the two typically developing groups did not display any differences in terms of performance. In addition, the authors did not find a significant correlation between IQ scores and performance on the task for the HFA group, but IQ did correlate with idiomatic comprehension and age for the two control groups. It is important to mention that this study used idioms out of context. The fact that the idioms were presented without a supportive context means that the participants had to rely exclusively on a semantic analysis or retrieval. Thus, the study design might have introduced an additional problem and a potential confound between expression familiarity/frequency and decomposability. In that study, Vogindroukas & Zikopoulou [26] confirm that children with AS/HFA have a tendency to make literal interpretations, but that the difficulty in idiom comprehension is definitely not due to an intellectual deficit.

Whyte et al [27] addressed what factors predict performance on idiom comprehension in highly-verbal children with autism in comparison to an age-matched control group and a control group matched on grammar-age equivalence. This study focused specifically on the contribution of syntax competence and advanced Theory of Mind (ToM) abilities to idiom comprehension in autism. The results document that children with autism indeed perform more poorly compared to age-matched controls, but that there is no significant difference with the syntax competence matched group. Furthermore, while advanced ToM ability did not predict performance in the control groups, it correlated with idiom comprehension in the participants with autism.

Lee et al [28] tested Korean high-functioning children with autism compared to similarly aged children with Attention Deficit Hyperactivity Disorder (ADHD) and a control group of typically developing children. The test design included matching idioms to a picture, which either described the figurative meaning of the expression or the literal interpretation. Non-figurative sentences paired with their literal interpretation were used as a base-line condition. This study found that both the children with autism and the ADHD group performed significantly worse than controls on the two idiom conditions, and especially in the condition where the idiom was paired with its literal interpretation. No differences were observed on the non-figurative expressions.

Norbury [29] investigated idiom comprehension in context in children with communication disorders and age-matched peers. Children belonging to the communication disorders group were selected to represent a spectrum of communication difficulty, including Specific Language Impairment (SLI), Pragmatic Language Impairment (PLI) (with or without presence of autistic symptoms), autistic disorder, and Asperger syndrome. An idiom definition task was chosen to measure idiom comprehension in context. The main finding of that study was that children with language and communication impairments benefit from context in interpreting unfamiliar idioms. However, children with deficits in structural language did not benefit from context as much as typically developing peers. Furthermore, children with autism spectrum disorder (ASD) symptoms and pragmatic impairments, but without concomitant structural language deficits, were not impaired to the same degree in their idiom understanding and were able to use context as effectively as their peers. Importantly, language ability was one of the most significant predictors of idiom understanding. These findings highlight the role of language skills as a key determinant in the use of context to aid idiom comprehension, and further suggest that contextual cues can provide sufficient information to derive at least a cursory understanding of new idiomatic expressions.

### Factors in idioms processing

Previous research has documented that idioms are more easily processed in the presence of supportive context ([16]; see Vulchanova et al [10] for a review). Findings from several studies suggest that the main role of context is to provide semantic support for decoding the target (appropriate) meaning of a sentence or an expression [17, 30–33]. In contrast, it is widely argued that individuals with autism are impaired in processing ambiguous linguistic information in context [34–36]. In addition, they often fail to attach context to their memories, and are specifically impaired in processing the social aspects of contextual information [37], while Ozonoff & Miller [38] demonstrate deviant use of context. However, the evidence concerning a contextual deficit is somewhat controversial. Thus, other studies have shown that competence at making use of context depends on language status, and children with higher verbal age can perform at the level of controls [39]. Also, success in that group may depend on the exact position of the ambiguous word (homograph) in the sentence. López & Leekam [36] found that, even though children with ASD performed worse than controls, they were more successful with homographs occurring later in the sentence (middle or end), suggesting sensitivity to context also in that group.

A study by Brock, Norbury, Einav & Nation [40] showed that participants with autism display a tendency similar to controls to use predictions based on the meaning of the lexical verb, as reflected in an increase in looks to the picture matching the object of the verb. At the same time both groups were less likely to be distracted by a phonological competitor of the object word. This tendency, however, was mediated by language status in the autism group. In that study context was restricted to the level of the sentence, with a focus on the information encoded in the lexical verb and the ability to use that information to successfully orient to the possible object. Also, Frith [41] argues that individuals with autism need a concrete context to be able to understand words and expressions, and Saldaña & Frith [42] document that children with autism have a normal reduction in reading times for expressions which are congruent with previous events. Given this evidence, we expect that context will not place any additional burden on the interpretation of the stimuli used in the current design.

Idiomatic expressions vary in degree of transparency/decomposability, and it seems clear that the more transparent the expression, the easier it is to understand, and conversely, the more opaque, the more difficult. A number of studies [43–44] demonstrate that the lexical makeup of idioms is a matter of degree and largely depends on the idiom’s compositional properties, and that idioms are accessed differentially according to their degree of syntactic frozenness. These authors thus claim that idioms do not form a unique class of linguistic items, but rather share properties with “more” literal language. Following this tradition, and with Titone & Connine [7], we expect that comprehension difficulty will be a direct function of the transparency/decomposability of the idiom [31, 33].

It can be assumed that the ability to understand idioms relies on the competence to process these expressions beyond the literal interpretation of individual words. This means that understanding idiomatic expressions demands making inferences, as well as integrating contextual information from both verbal and non-verbal sources. In our daily life, our knowledge about the world around us is based on the semantic and conceptual information gathered through our senses, implying that we perceive the world through different modalities [45]. Consequently, it seems logical to expect that some of the difficulties that individuals with autism demonstrate in figurative language may be due to a more generic deficit in multimodal integration. Indeed, problems with multi-sensory information have been documented in autism, and specifically at speech-specific multisensory integration [46]. Irwin, Tornatore, Brancazio & Whalen suggest that there are fundamental differences in audio-visual integration in children and adolescents with autism in comparison to typical peers [47]. Furthemore, individuals with autism have been shown to present with a reduced McGurk effect in the processing of audio-visual speech [48]. It has also been suggested that the neurodevelopmental abnormalities in autism may be due to poor information integration across brain regions [49]. Van Der Smagt, Engeland & Kemner [50] further suggest that any problems arising from integrating auditory and visual information must stem from higher processing stages, as is the case in processing complex linguistic material, and not low-level integration processes (e.g., at the level of individual sound/visual stimulus).

## Hypotheses

In this study, we investigate the interaction of linguistic and visual context in the interpretation of figurative expressions in highly-verbal individuals with autistic spectrum disorder compared to typically developing peers. The aim of this research is to test a more homogeneous sample of participants within the broad group of individuals with autism by focusing on the higher end of the spectrum (high functioning individuals with intact structural language) in two homogeneous chronological age samples. Thus, we are interested in establishing a potential developmental trajectory, albeit through a cross-sectional design.

Consistent with the evidence from previous research, we expected to find effects of age and group. In particular, we hypothesized that the two groups with autism will perform significantly worse than the age- and verbal abilities-matched control groups. Specifically, we expected to find higher reaction times (RTs) and error means in the participants with autism compared to their typically developing peers, despite an improvement with age, as observed in the case of metaphors in a recent study (Chahboun et al., [25]). Furthermore, it can be expected that, since individuals with HFA have a tendency for literal interpretation, they will have difficulties in appreciating the figurative nature of non-literal language and more often select interpretations that match the literal meaning of the expression. Given that figurative language skills develop constantly through childhood, we also expect that younger participants (the two children groups) will perform at a lower level than the young adult groups.

While cross-modal tasks, which require the integration of information from different modalities may not present a problem for typically developing participants, they may tax processing in individuals with autism and make the task more demanding. Concerning the presence of visually supported meaning (pictures that matched the interpretation of the idioms), it can be expected that visual context may facilitate the processing of the figurative meaning, or, at least, will not present an additional problem, as suggested by López & Leekam [36] and indicated by the results in Brock et al [40], also consistent with the findings in Norbury about the facilitatory role of context [29]. Alternatively, it may be the case that the presence of both auditory and visual information might rather present a problem, as a result of the demand for information integration in the face of documented problems in that domain in autism. Thus, we anticipated higher error rates and RTs in the condition when stimuli were presented in a cross-modal fashion (auditory stimulus/visual target) in the groups with autism, and the absence of such an effect in the control groups.

One of the most important criteria in the processing of figurative language is expression transparency. We expected that degree of transparency would impact both on reaction latencies and accuracy in both participants with autism and controls. More specifically, the most transparent category, the one labelled *biological idioms* would be easiest to process, while *cultural idioms*, which are more highly conventionalised and less transparent would, in comparison, present greater difficulty, especially for the child participants and participants with autism. We also expected novel metaphors and *instructive idioms* to be more easily processed than the less transparent *cultural idioms*.

## Method

### Participants

Two age groups of HFA (*n* = 45) and typically developing (TD) controls (*n* = 39) were recruited, all native speakers of Spanish. The first age group included children in the age range ten to twelve years. The second age group included young adults from sixteen to twenty-two years old (see Table 1 for the participants’ descriptive and demographic data). Participants and their legal guardians (usually the parents) provided written informed consent for entry into the study according of the declaration of Helsinki principles. The study was approved by the Andalusian Biomedical Research Ethics Committee. The diagnosis of HFA was confirmed with the Autism Diagnostic Observation Schedule (ADOS) [51] (Children, $\bar{x}$ *=* 10,8; Young adults, $\bar{x}$ *=* 12,3).

**Table 1:** Mean (and standard deviation) of background measures for each age and group. HFA (high functioning autism), FSIQ (intelligence quotient, Full Scale score), VCI (verbal comprehension index), RG (receptive grammar scores), RC (reading comprehension scores). Note: *p* < .05 for scores with the same superscript.

	**Children**		**Adults**	
	HFA	Control	HFA	Control
**n**	25	19	20	20
**Age (months, SD)**	11,3 ±0,96	11,9±0,89	18,1±1,65	18,3±1,92
**Gender (M)**	21	17	17	14
**FSIQ**	110,71±14,58	105,76±11,56	108,3±13,39	118,34±8,46
**VCI**	118,7±17,47	107,4±15,03	122,62±16,71	117,63±12,97
**RG**	72,72±5,56	74,31±4,30	74,40±3,53^a^	78,42±1,74 ^a^
**RC**	0,58±0,17	0,59±0,16	0,63±0,15	0,72±0,12

The individuals with HFA and their typically developing peers were matched on age, gender and the verbal comprehension based on the Wechsler scale (WISC-IV [52] or WAIS-IV [53] were used, depending on the chronological age of the participant). The Verbal Comprehension Index (VCI) was used as an overall measure of verbal reasoning and includes measures of semantic/conceptual reasoning (similarities and vocabulary), and verbal comprehension. The descriptive statistics are given in Table 1. The matching of the groups was based on the Wilcoxon Test and the smallest *p* value was *p* = .222 suggesting that there were no significant differences observed between the groups (See Table 1). We also considered reading comprehension scores and receptive grammar (based on CEG, which is the Spanish adaptation of TROG [54]) as background measures to ensure our participants display scores within the norm (see Table 1). No differences were observed between participants for reading comprehension. However, despite all our participants being within the norm for receptive grammar, differences were observed between the two groups of young adult participants. For this reason, we included receptive grammar as a co-variate in the analyses of the data.

### Apparatus and stimuli

In a pilot study with 50 adults, all native speakers of Spanish, we determined the degree of familiarity and frequency of the figurative expressions to be used in the main study. We selected 124 idioms and instructive expressions and 20 metaphors, for which participants in the pilot study had to determine: 1. “Do you know this expression?” 2. “If yes, do you know what it means?” 3. “Do you use this expression yourself?”. Participants were asked to answer truthfully, and to take into account that there were no wrong answers. We established ratings for each expression by assigning numerical values to the answers (1 for *yes* and 0 for *no*) and averaging the scores from the 3 questions. The idiomatic expressions which received a rating of over 0.80 were selected for the study and classified into three main categories: a/ biological idioms; b/ cultural idioms; and c/ instructive expressions, as used in Vulchanova et al. [8]. For the first two categories they followed the typology adopted in Penttilä, Nenonen & Niemi [55], in turn inspired by Searle’s idea of deep background (the human biological nature) vs. local background (local cultural practices), to account for the grounding of language in human experience and practice. The stimuli in these two categories were selected by 2 independent expert linguist raters (91% interrater agreement). Examples of biologically-based idioms are e.g., *estar con el agua hasta el cuello* (to be with water up to the neck); or *venir como anillo al dedo* (to come as ring to the finger), and of culturally-based idioms, *e*.*g estar como una cabra* (to be like a goat). The difference between the two categories is that, while biological idioms are derived from human (bodily) experience and interaction with the environment, and as such, tend to be more transparent and available to compositional parsing, cultural idioms are more idiosyncratic, tend to vary from one culture to another, and are, in general, less transparent. Our third category were instructive expressions (i.e., proverbs), whose meaning can be computed on-line, by drawing inferences on the basis of their individual constituent phrases (e.g., *Las mentiras tienen las patas cortas—*Lies have short legs—). The inclusion of this category was dictated by the need to compare such expressions, whose interpretation may be more directly available in appropriate context, to the comprehension of idioms. Finally, novel metaphors were included as a category that may be assumed to be less frozen/fixed, and as such more readily interpretable. From the pilot study, we selected for inclusion in the novel metaphors category those metaphors which earned a rating below .20 on the familiarity and frequency scale (e.g.: *Estas flotando en el aire*—You are floating in the air).

**Fig 1:**
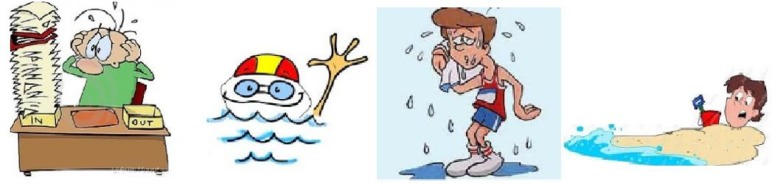
Images for the idiom “*estar con el agua hasta el cuello*!*”* (lit.: be with water up to the neck/fig.: drown in work)

The study was designed as a sentence-picture matching task (38 visual—presented as text—and 38 auditory expressions (a total of 76 expressions—20 biological idioms/20 cultural idioms/16 instructive expressions/20 metaphors) randomized and counterbalanced between groups and participants). Each expression was linked to four possible images: One reflecting the figurative target meaning of the expression, a second image reflecting the literal meaning, a third image corresponding to a figurative meaning, but not the target one, and finally, a distractor as forth image (Cf. Fig 1). Each expression was presented in a short appropriate context (See S1 Appendix for examples). Thus, the study design included both verbal (presented as text/discourse) and visual (pictures) context.

Matlab (R2014b) (matrix laboratory) was used as a multiparadigm numerical programming language to design, build and run the experiment.

### Procedure

In this experiment, we used visual stimuli (images and sentences) that reflected literal meanings and idiomatic meanings. First, a fixation point (+) appeared for 500ms. Participants then saw on the screen the idiom or metaphor. Depending on the experimental block, the participant heard the expression in context via loud-speakers or read it in context in a short text presented on the computer screen orthographically. Thus, the condition in which stimuli were presented auditorily presented a cross-modal environment whereby participants had to select a visual target which matched a figurative expression they had heard via the loud-speakers. In the auditory experimental block, first the figurative expression appeared on the computer screen followed by a blank screen, while the expression in appropriate context were being presented auditorily. In the text (orthographic) condition both stimulus (expression and context) and target picture were all in the same (visual) modality. First the expression appeared on the screen, and was then followed by the context. Then, in both experimental conditions a fixation point (+) reappeared in the middle of the screen followed by four images in each corner, each one reflecting the figurative meaning (target) meaning, the literal meaning, figurative, but not target, meaning, and a distractor image. The position of presenting the images was counterbalanced between participants and between the modality of presenting the context and the order of the expressions. The participant’s task was to click on the stimulus he/ she considered most appropriate for the meaning of the expression. Accuracy and reaction times were collected to determine the ease of processing.

## Results

The data of both the control and experimental groups (n = 84) were analysed with R [56, 57]; and the lme4, afex and lmerTest packages [58]. We used two performance measures in the study; reaction time which reveals the ease with which the figurative expression is processed, and accuracy which essentially measures the ability to link the figurative expression to its target image. We ran linear mixed models on reaction times and generalized linear mixed models on accuracy, including the variables of Group (ASD vs. control), Age (Children vs. Young Adults), Modality (Visual vs Auditory), and Type of Expression (Biological, Cultural, Proverbs, and Novel Metaphors). We report relevant F and p-values below (based on the Satterthwaite approximation for degrees of freedom) for reaction times and Chi square and p-values for accuracy.

We ran four analyses. The first one, the reaction time analysis, provided us with a general overview of the ease with which the participants responded to the different experimental manipulations in the study. The next analyses explore the ability to identify the correct target compared to other options. Thus, a second model provided us with participants’ accuracy and compared overall target and non-target responses. The third and fourth specifically explore correct to other specific non-appropriate responses. The third analysis, called “comparison between figurative target responses and literal responses”, provides a comparison between responses to target images and images corresponding to the literal meaning. Finally, our fourth model is based on a comparison between figurative target responses and figurative non-target responses. This model only includes responses to images reflecting target meanings and responses to images which reflected a figurative albeit not target meaning.

### Reaction time analyses

Reaction times were analysed using Log(RT) (ms) as a measure rather than reaction times to correct for right skewedness of the reaction time data. Only correct responses are included in the analyses. We also excluded extremely fast or slow responses. We excluded two trials with reaction times longer than 18s and we calculated the Z value of Speed (Log(RT) (ms)) for each participant (Z_p_) and for each item per age and group (Z_i_). If the sum of the squares of the Z values was smaller or equal than 8 (Z_p_^2^+Z_i_^2^ ≤ 8), the trial was included, and values bigger than 8 were excluded.

An overall linear mixed model analysis on Log (RT) (ms) performance has been done with R, which provided us with the best fitting model by step-wise backward selection based on the Akaike Information Criterion (See S2 Appendix). We were left with a model including a three-way interaction of group, modality and type of expression, a two-way interaction of age and type of expression, and all lower effects. Also included in the model were the by-subject random intercepts and slopes for modality and by-item random intercepts and slopes for Age and Group.

**Fig 2:**
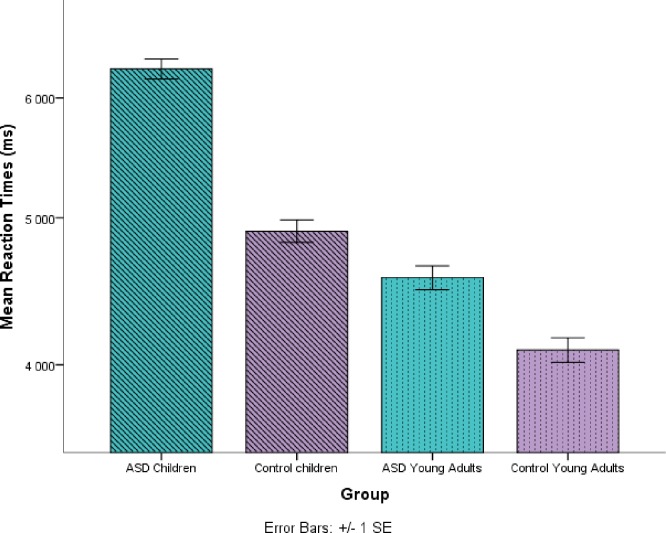
Average reaction times (ms) for each age (children/young adults) and group (control/HFA). Error bars denote one standard error of the mean.

**Fig 3:**
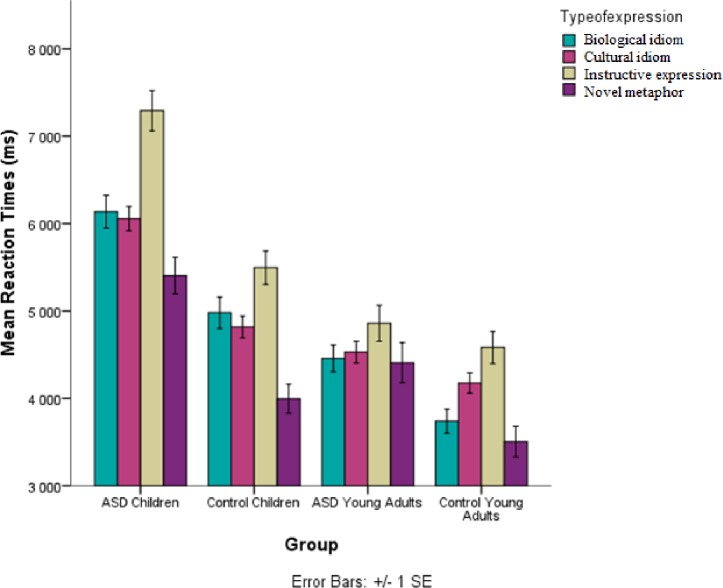
Average reaction times (RTs) for each age (children/young adults) and group (control/HFA) for each type of expression. Error bars denote one standard error of the mean.

The overall model reveals that there are differences in reaction latency for Age (children/young adults) (*F* (1, 82.64) = 20.38, *p* < .001) (Satterthwaite approximation for degrees of freedom) and Group (control/HFA) (*F* (1, 86.85) = 10.64, *p* = .001), with slower responses by children and individuals with autism (see Fig 2). Furthermore, an interaction between age and type of expression was found (*F* (3, 66.11) = 2.903, *p* = .041) (see Fig 3). A multiple comparison with Tukey contrasts showed that the interaction is due to differences between different types of expressions in children, but not adults. Here, a significant difference was observed between novel metaphors and instructive expressions (*p* = .0014). In addition, a marginally significant difference between cultural idioms and novel metaphors was observed (*p* = .07) showing that instructive expressions were the most demanding and difficult to process for the children.

**Fig 4:**
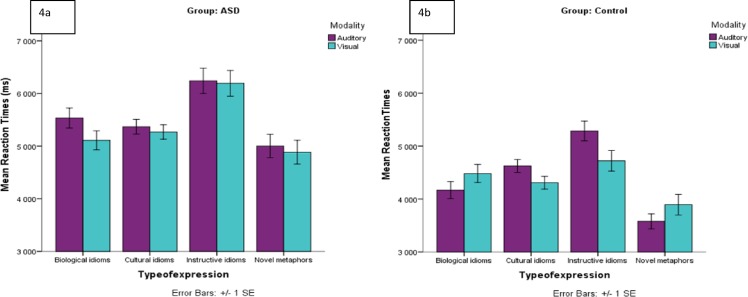
**4a, 4b. Average reaction times (ms) group (control/HFA) type of expression and modality**. Error bars denote one standard error of the mean.

As we have pointed out, a three-way interaction between type of expression, group and modality was observed (*p* < .001) (see Fig 4A and 4B). In order to establish the origin of the three-way interaction, additional multiple comparisons with Tukey contrasts were run. For the HFA group in the auditory modality, no significant differences were observed between different types of expressions. However, for the visual modality, an almost significant difference was observed between instructive expressions and novel metaphors (*p* = .0501), with novel metaphors processed faster than instructive expressions. For the control group in the auditory modality, significant differences were observed between instructive expressions and biological idioms (*p* = .013), with slower responses in the instructive expressions category, and between novel metaphors and cultural idioms (*p* = .033), with faster responses in the novel metaphor category, and between novel metaphors and instructive expressions (*p* < .001), with faster responses for novel metaphors. No significant differences were observed in the visual modality in the control group between different types of expressions. These results suggest a different interaction pattern between participants with autism and controls. While for controls there is differential processing depending on type of expression in the oral language modality, with more transparent expressions processed faster, this pattern is absent in participants with autism.

Moreover, we explored the differences between groups and modalities for each type of expression. For biological idioms, while the control group was faster in the auditory modality, the HFA group was faster in the visual modality (*p* = .026), but always significantly slower than controls (*p* = .001). For cultural idioms, the HFA group was significantly slower than controls (*p* = .001). In addition, no modality differences were observed in the HFA group for this type of expression, while the control group were faster in the visual modality (*p* = .02). For instructive expressions, the control group was significantly slower in the auditory modality (*p* = .01), while the HFA group showed no modality preference in this category and was slower in comparison to the control group overall in this type of expression (*p* = .015). Finally, for the novel metaphor category, again the HFA group was much slower overall (*p* < .001), and showed no modality differences, while the control group showed faster responses in the auditory modality (*p* = .01). To sum up those results, with increase of expression non-transparency, the advantage of modality changes only for controls. Thus, for controls transparent expressions are processed faster in the oral modality and less transparent expressions are processed faster in the orthographic modality. No such trend is observed for the participants with autism. These results suggest a different processing pattern in autism.

### Comparison between figurative target responses and non-target responses

An overall generalized linear mixed model analysis on ACC (mean errors) performance has been done with R, which provided us with the best fitting model by step-wise backward selection based on the Akaike Information Criterion. We were left with a model including a two-way interaction of age and group and main effects. Also included in the model were the by-subject random intercepts and slopes for modality and by-item random intercepts and slopes for Age and Group.

**Fig 5:**
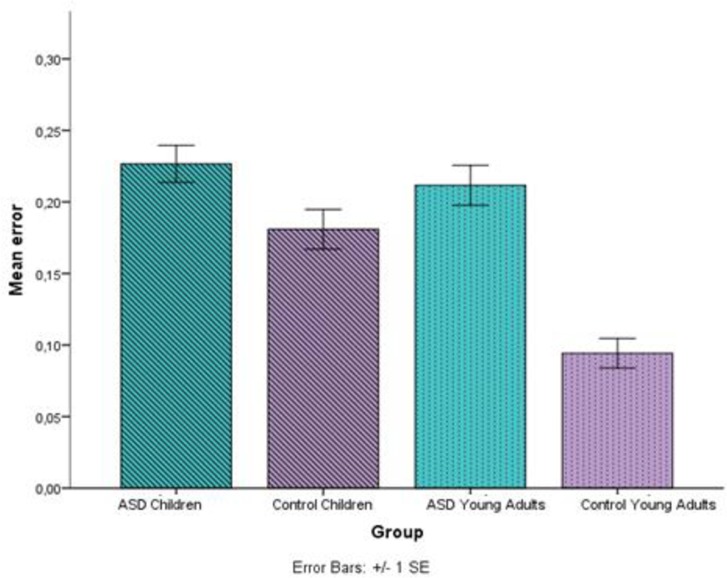
Average response accuracy (mean errors) for each age (children/young adults) and group (control/HFA). Error bars denote one standard error of the mean.

The overall model thus reveals differences in accuracy in Age (children/young adults) (*χ^2^* (1, 23) = 5.73, *p* = .0016) and Group (control/HFA) (χ^2^ (1, 23) = 11.21, *p* < .001), with more errors by children and individuals with autism (see Fig 5). In addition, a main effect of type of expression was observed as well (*χ^2^* (1, 21) = 8.49, *p* = .036). Multiple Comparisons of Means with Tukey contrasts (See S3 Appendix) revealed that this effect was due to marginally significant differences between instructive expressions and biological idioms (*p* = .07), and between instructive expressions and novel metaphors (*p* = .06), with more errors in the instructive expressions category.

Moreover, a two-way interaction between age and group was observed (*χ^2^* (1, 23) = 4.98, *p* = .025). Multiple Comparisons of Means with Tukey contrasts (See S3 Appendix) revealed that the interaction was due mainly to the differences between young adults (HFA and control) (*p* < .001), and between the two control groups (children and young adults) (*p* = .01). No differences were observed between the two HFA groups (*p* = 0.99) or children (HFA and control) (*p* = 0.74). These results suggest that participants with autism perform at the same level regardless of age.

### Comparison between figurative target responses and literal responses

In order to establish responses to target figurative meaning contra literal interpretations, we re-ran the model, while excluding non-target figurative, and distractor responses (See S4 Appendix). In this model, Target responses were scored with 1 and literal responses were scored with 2. The model includes a significant three-way interaction between modality, age and group (control/HFA), and all lower interactions and main effects. Also included in the model were the by-subject random intercepts and slopes for modality and by-item random intercepts and slopes for Age and Group.

**Fig 6:**
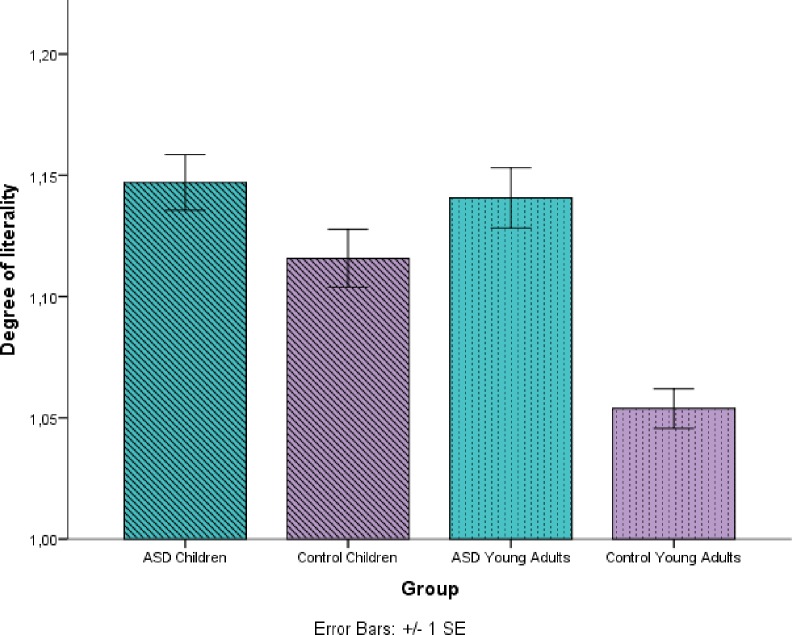
Average literality for each age (children/young adults) and group (control/HFA). Target responses were scored with 1 and literal responses were scored with 2. Higher means indicate more literal responses. Error bars denote one standard error of the mean.

This model reveals a main effect of group (control/HFA) ((*χ^2^* (1, 26) = 5.22, *p* = .022), with more literal responses by children and young adults with autism and a marginally significant difference in accuracy between Age (children/young adults) ((*χ^2^* (1, 26) = 3.51, *p* = .06) (see Fig 6). In addition, a main effect of type of expression was observed (*χ^2^* (1, 24) = 10.37, *p* = .015). Multiple Comparisons of Means with Tukey contrasts (See S4 Appendix) revealed that this effect was due to a difference between instructive expressions and novel metaphors (*p* = .02), with more literal responses in the instructive expressions category, and to a marginally significant difference between cultural idioms and novel metaphors, with more literal responses in the cultural idioms category. These results suggest that less transparent expressions are likely to trigger more literal responses.

Furthermore, a two-way interaction between age and group was observed (*χ^2^* (1, 26) = 4.89, *p* = .02). We ran multiple comparisons of means with Tukey contrasts which revealed that this interaction was due to a significant difference between control young adults and HFA young adults (*p* = .015), with the young adults with autism giving more literal responses than their respective typically developing peers. Also, the difference between both ages of control participants was significant (*p* = .03), where the young adults detected the figurative target image more accurately. No differences were observed between the two HFA groups (*p* = 0.99) or children (HFA and control) (*p* = 0.99). These results suggest that children and participants with autism are more likely to provide literal responses.

**Fig 7:**
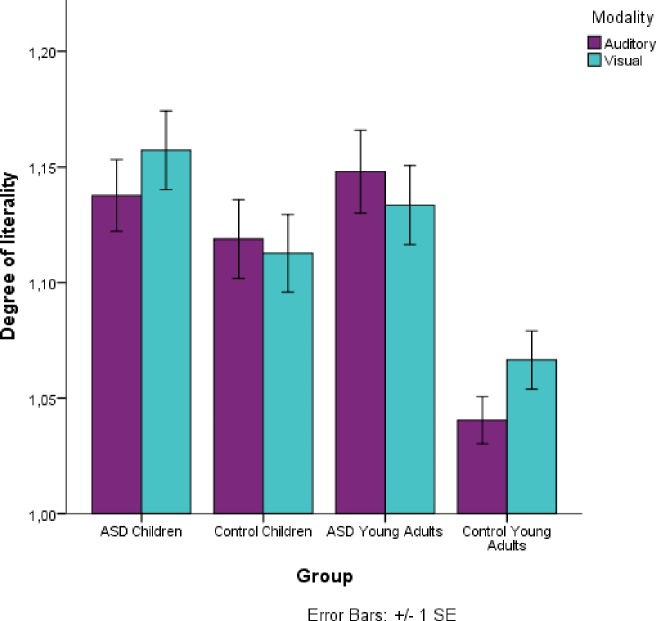
Average literality for each age (children/young adults) and group (control/HFA) and modality (visual/auditory). Target responses were scored with 1 and literal responses were scored with 2. Higher means indicate more literal responses. Error bars denote one standard error of the mean.

A three-way interaction between age, group and modality was observed (*χ^2^* (1, 26) = 3.97, *p* = 0.046) (see Fig 7). Again, a multiple comparison of means with Tukey contrasts showed no significant effects in the visual modality in any of the age groups, but clear differences in the auditory modality between the control young adults and the young adults with autism (*p* = 0.001), where the older groups of participants had inverted patters of responses. The young adults with HFA were less literal in the visual modality than the auditory modality, while the control young adults were better in the auditory modality, suggesting a differential pattern in response. Moreover, a marginally significant difference was observed between the control children and the control young adults (*p =* .*055)*, where the children did not show modality differences, but the older group was better in the auditory modality.

### Comparison between figurative target responses and figurative non-target responses

**Fig 8:**
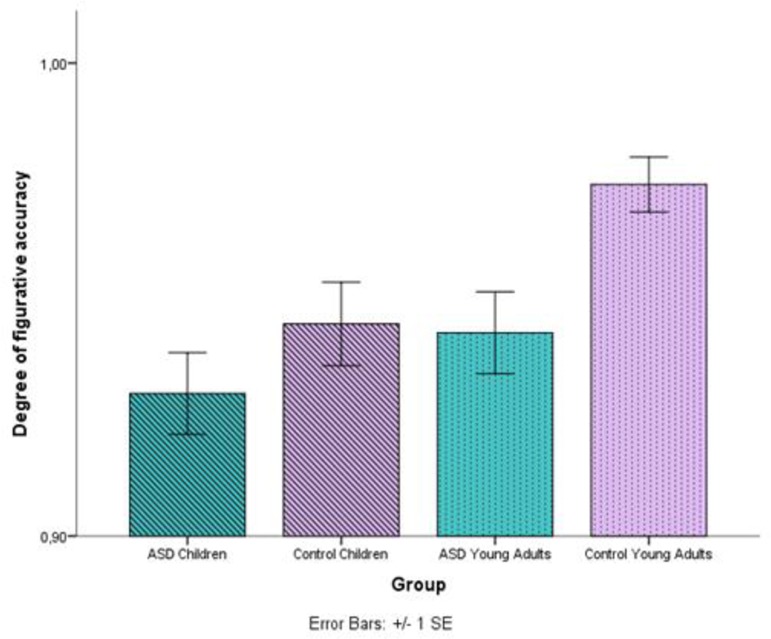
Average accuracy in figurative meanings for each age (children/young adults) and group (control/HFA) and modality (visual/auditory). Target responses were scored with 1 and literal responses were scored with 0. Higher means indicate more accurate responses. Error bars denote one standard error of the mean.

The final part of the analyses compared figurative target responses and figurative non-target ones. Therefore, we ran the model again without responses to the literal image and responses to the distractor (See S5 Appendix). From the overall model it is clear again, that there are differences in accuracy between groups (control/HFA) (*χ^2^* (1, 11) = 6.49, *p* = .01), with more errors by individuals with autism. Additionally, a main effect of age (children/young adults) was found as well (*χ^2^* (1, 11) = 12.92, *p* < .001 and (see Fig 8), with more errors in the two younger groups. These results suggest that figurative language comprehension is not yet stabilized and still developing in the child participants. No other significant effects were found in this comparison. Despite this, figurative non-target responses only appeared in very few instances (4.5% of the responses in comparison with 81.8% for figurative target ones). Consequently, the comparison between figurative target and figurative non-target responses shows a very small error range.

### General linear model—Receptive grammar as a covariate

We were also interested in the extent to which the difference between the two young adult groups on receptive grammar influenced these groups’ performance on the tasks. Repeated measures ANOVA were run in SPSS [59], where we considered age and group as between subject factors, modality and type of expression as within subject variables and receptive grammar as a covariate. Bonferroni confidence interval adjustment was considered and observed power was calculated in this analysis (See S6 Appendix). We followed the same procedure for all the previously considered dependent variables (reaction time, overall accuracy, scores for target and literal responses, and scores for target and figurative non target responses).

Receptive grammar as a covariate did not affect in any way any of the models and none of the main effects or interactions changed with the inclusion of receptive grammar scores as a covariate in the regression model. We report the power effects analyses in S7 Appendix.

## Discussion

Our main hypothesis was that we would find poorer performance on figurative language processing by children and the participants with autism compared to their peers, carefully matched on age, non-verbal IQ, and verbal comprehension. Consistent with our hypotheses, the analyses of reaction times in the current study found a main effect of age and group, with slower responses for children and participants with autism. While no significant difference was observed between the two children’s groups on a test of receptive grammar, there was a difference between the two young adult groups of participants, and oral language differences could have explained the different performance between these groups. However, the fact that our results were not affected by the inclusion of receptive grammar scores as a co-variate in the analysis, ensures that the obtained results were not due to a structural language problem (e.g., in receptive grammar), but to the experimental manipulation.

These analyses also revealed that two types of expressions were particularly difficult for children, cultural idioms and instructive expressions (proverbs), while novel metaphors were processed much faster. These results suggest that the impact of expression transparency tends to diminish with age and as figurative skills stabilize in young adulthood.

The analyses of accuracy revealed a similar pattern: more errors were found in the two child groups and in the two groups with autism. Here a main effect of type of expression was also observed, whereby instructive expressions emerged as the most difficult category to process in comparison to both novel metaphors and biological idioms. The analyses of accuracy also revealed that participants with autism were significantly more likely than controls to select the literal interpretation of the figurative expression, as represented in a visual image.

In this study, we hypothesized that the participants with autism would display a relative weakness in performance on idiomatic language, despite intact structural language skills. Although individuals with autism obtain high accuracy scores and can obviously solve the task, the current findings confirm that the adult individuals with autism are not reaching an equivalent level of performance to typically developing matched controls, both with regard to accuracy and reaction time. These results are consistent with results from the few previous studies of idiom comprehension in autism, where the participants with HFA performed more poorly and more slowly than their typically developing matched peers, suggesting a different pattern of processing figurative language in general, and idiomatic competences, in particular. These results are also consistent with the finding that the contrast in ability in autism in comparison to controls is more evident on the higher end of the spectrum [60].

The current results, however, contradict findings of comparable semantic and language processing skills in studies where the controls and participants with autism are matched on structural language skills [27,40]. The participants in the current study were highly-verbal individuals with autism carefully matched to the control groups on IQ and verbal skills, including semantic, and oral and reading comprehension skills. The current results thus challenge the idea that the problems in figurative language in autism arise exclusively from verbal status and language skills, also consistent with results in Eigsti, de Marchena, Schuh & Kelley [61] and Olofson et al. [62] and the review in Vulchanova et al. [10]. More importantly, both the reaction latencies and accuracy analyses reveal a different processing pattern in the interaction between modality (auditory/visual) and type of expression, especially evident in the young adult groups. This pattern of responses is suggestive of different processing or perhaps alternative strategies for the comprehension of figurative language in autism, despite overall high accuracy.

We consider that this different pattern of processing is due, in part, to a delayed trajectory in developing the necessary skills to process figurative language. This is clearly seen in the similar response pattern of the young adults with autism and the control children on both accuracy and reaction speed. This may suggest that rather than an inability to process figurative language or idioms altogether, in this case, there is a delay in the ability to detect such expressions, and to differentiate between the different categories of figurative language. These results support the findings from another study with the same participants on metaphorical language [25]. Yet, even though participants with autism seem to improve with age in terms of reaction time, suggesting faster processing, they still do not reach the same levels of performance as control matched peers. The improvement with age does not only apply to the individuals with HFA, but also to the control groups, which is consistent with Nippold & Duthie [63], Levorato & Cacciari [64] and Vulchanova et al [8], and our original hypothesis, but not with Vogindroukas & Zikopoulou [26], who, surprisingly, did not find any reliable difference in performance between the younger and older controls.

Furthermore, our study explored the impact of the nature and the transparency of the type of figurative expression. In the study we included biological idioms, which have been argued to be more closely grounded in human experience and interaction with the environment (Searle’s *deep background*). We expected such expressions to be more readily accessible and more decomposable, despite their idiomatic nature, compared to cultural idioms, also for the participants with autism. In line with Chahboun et al [25], we also expected that novel metaphors would be easier to process. These expectations were confirmed. Even though the participants with autism were significantly delayed in comparison to controls, they displayed similar patterns of difficulties and ease across the test categories as the control groups. Biological idioms and novel metaphors were the easiest to process. We reason that these two categories are the most transparent, and their meaning can be easily inferred from the context, while cultural idioms are less transparent and more “frozen” [65].

These findings are consistent with Vulchanova et al. [8] and Vega-Moreno [17] who claim that inferential skills are not only involved, but mandatory in idiom comprehension. In our study, the figurative expressions were elicited based on a pilot, where we selected the most familiar and frequent expressions for the idioms category, and the least frequent (most novel) for the metaphors category. The current results confirm that novel metaphors were easier to process compared to less transparent expressions, such as idioms. Our findings are partly consistent with Giora, Gazal, Goldstein, Fein, & Stringaris [66], who found that familiarity/novelty of the expression were reliable predictors of performance in both typically developing individuals and individuals with HFA. The current results are also consistent with the findings in Norbury [29], where participants with autism were facilitated by the presence of context in the comprehension of novel idioms and performed at levels similar to controls. The current findings, however, contradict the results obtained by Mashal & Kasirer [24]. These authors found that the most novel figurative expressions were the most difficult to process in both adults and children with HFA. However, this difference might be due to experimental design and choice of experimental stimuli, as is often the case in research on figurative language.

Contrary to our expectations, instructive expressions (proverbs) were the most difficult category to process, along with cultural idioms. We included this category in the design because of the expectation that they may not be stored in the mental lexicon, but rather rely on the immediate activation of richer “memory organization packages” [21,67], thus allowing more readily for interpretations on the fly when encountered in appropriate context. This expectation was not confirmed, however. Moreover, both children and participants with autism were significantly more likely to select the literal interpretations of both instructive expressions and cultural idioms.

These results support the findings in Honeck, Welge & Temple [68] concerning the processing of proverbs, where participants were faster to make appropriateness judgements when the proverb was related literally to the context compared to when it was related figuratively. These authors interpret the findings as supporting a multi-stage model of proverb comprehension, with literal interpretations becoming available faster than figurative ones. The current results suggest that instructive expressions (proverbs) indeed require some ability to think abstractly [20] and cannot be interpreted so readily based on their constituent words. We also find evidence that this skill takes time to acquire, as evident in the child participants. We also speculate that the more culturally-based an expression is, the more reliant it is on learning it in social interaction, thus consistent with the limitations documented in this domain in autism.

Regarding the effect of modality, we hypothesized that the control group would not display dissociations between the visual and auditory presentation modalities, while for the participants with autism we expected certain problems when the expressions were presented auditorily and had to be matched to a visual stimulus (a picture) in the cross-modal condition. This was confirmed, but only for the most transparent expressions (biological idioms and novel metaphors). The control groups performed better when biological idioms and novel metaphors were presented in the auditory modality. Thus, it appears that the control group have an advantage for oral language, and can easily integrate oral stimuli with visual contextual support (the images). This was not the case for the participants with autism, and it could suggest a a difficulty even in highly-verbal individuals with autism in multimodal information integration, even when the idiomatic expressions are decomposable (cf. also Ozonoff & Miller, [38]). These differential responses to modality in the autism group could be indicating the involvement of different processes in the resolution of the idioms, or different approaches when attempting to respond to this task.

These findings point to the possibility of a problem of simultaneous processing in both modalities in this population. An additional possibility may be that there are residual oral language problems also in individuals with intact structural language skills. This is consistent with Eigsti [69], where the problems in ASD are attributed to low-level impairments in generic cognitive and processing mechanisms. Alternatively, it can be assumed that “seeing” text is an advantage, especially in the groups with autism. Indeed, there is evidence that orthographic decoding is a strength in autism and this may, in turn, lead to better comprehension outcomes, if a simple view of reading is adopted [70].

Another interesting result was the difference in degree of literalness observed in response accuracy. As expected, the younger participants and participants with autism interpreted the stimuli more often literally than the older and the control groups. Figurative language competence takes longer to develop and requires a number of other skills (inferencing, discourse skills; [71]), and the older we are, the more resources for processing we have. In the case of participants with autism, both children and young adults were very literal and did not appear to improve in this respect with age (the two age groups in the study performed similarly). This was not the case for the control groups. For children presentation modality did not affect response literalness, while for the older participants there was an advantage in the auditory modality, suggesting that idiom comprehension most probably resides in mature oral language skills. The results of the participants with autism are also consistent with other research suggesting a tendency for literal (compositional) interpretation in autism [26,72].

The overall results of this study support our hypothesis that idiomatic expressions require more competences than just good structural language. These clearly provide a necessary condition, but may not be sufficient. This is evidenced by the findings supporting the presence of a delay in the two groups with HFA. Additionally, the current study contributes to research studying the processing of idioms specifically, and figurative language, more broadly, in providing empirical evidence on how different categories of figurative expressions are processed in both highly verbal individuals with autism and typically developing individuals. We find support that expressions, which are more firmly grounded in human bodily experience (biological idioms), and are also more transparent (novel metaphors) are processed more readily, despite their idiomatic nature, regardless of participant group. Finally, we find evidence that young adult controls have a clear advantage in processing auditory language stimuli, most likely as a result of subtle differences in oral language skills. The problems observed in the participants with autism are suggestive of either multi-sensory information integration difficulty or simply a residual problem in oral language and need to be addressed in future research potentially leading also to better target intervention for that group.

## Supporting Information

S1 Appendix Examples of expressions in context. (DOCX)

S2 Appendix Overall model (Reaction times). (RTF)

S3 Appendix Overall model (Accuracy analysis: Target vs non-target). (RTF)

S4 Appendix Overall model (Accuracy analysis: Target vs literal responses). (RTF)

S5 Appendix Overall model (Accuracy analysis: Figurative target vs Figurative non-target responses). (RTF)

S6 Appendix General linear model analyses with receptive grammar as covariate. (DOCX)

S7 Appendix Observed powers. (DOCX)
