# Supplementary material for: Can You Play with Fire and Not Hurt Yourself? A Comparative Study in Figurative Language Comprehension between Individuals with and without Autism Spectrum Disorder
Source: PLoS One. 2016 Dec 30;11(12):e0168571. doi: 10.1371/journal.pone.0168571 (PMC5201294; doi:10.1371/journal.pone.0168571)
Supplement: S1 Appendix — (DOCX) [file pone.0168571.s001.docx]

**Appendix S1. Examples of expressions in context**

| Expression | Type | English translation | Context in Spanish | Context in English |
| --- | --- | --- | --- | --- |
| Estás flotando en el aire | Novel metaphor | You are floating in the air | En una clase, el profesor le dice a Juan: “Juan! Parece que estás flotando en el aire!” | In a classroom, the teacher tells Juan: “Juan! It looks like you are floating in the air!” |
| Estar con el agua hasta el cuello | Biological idiom | To be with water up to the neck (to be drowning in work) | Juan tiene muchísimo trabajo acumulado y no sabe por dónde empezar. Se siente con el agua hasta el cuello. | Juan has a lot of accumulated work and does not know where to start. He is up to his neck in it (water). |
| Estar como una cabra | Cultural idiom | To be like a goat (Mad as a march hare) | Juan es un científico que se arriesga con experimentos peligrosos. Sus vecinos dicen que está como una cabra. | Juan is a scientist who risks with dangerous experiments. His neighbors say he is like a goat. |
| Muerto el perro, se acabó la rabia | Instructive expression | Dead the dog, the rabies is gone (once the problem is solved. everything is fine) | A Juan le duele mucho la muela. María lo lleva al dentista. De camino de vuelta a casa, María le dice a Juan : “Muerto el perro se acabó la rabia”. | Juan has a tooth ache. María takes him to the dentist. On their way back home, María says: “Dead the dog, the rabies is gone”. |
