# Supplementary material for: Can You Play with Fire and Not Hurt Yourself? A Comparative Study in Figurative Language Comprehension between Individuals with and without Autism Spectrum Disorder
Source: PLoS One. 2016 Dec 30;11(12):e0168571. doi: 10.1371/journal.pone.0168571 (PMC5201294; doi:10.1371/journal.pone.0168571)
Supplement: S2 Appendix — (RTF) [file pone.0168571.s002.rtf]

Appendix S2. Overall model (Reaction times)

Fit: lme4::lmer(formula = log10(RT) ~ Age2 * Typeofexpression + Age2 * 
    Group2 + Group2 * Mod2 * Typeofexpression + (1 + Mod2 | part) + 
    (1 + Age2 * Group2 | Item), data = data)
Sum Sq Mean Sq NumDF   DenDF F.value    Pr(>F)    
Age                          0.34952 0.34952     1   82.64 20.3839 2.083e-05 ***
Typeofexpression             0.10478 0.03493     3   37.04  2.0369  0.125481    
Group                        0.18246 0.18246     1   86.85 10.6413  0.001581 ** 
Modality                     0.03973 0.03973     1   85.39  2.3171  0.131655    
Age:Typeofexpression         0.14934 0.04978     3   66.11  2.9032  0.041257 *  
Age:Group                    0.01153 0.01153     1   80.91  0.6725  0.414593    
Group:Modality               0.00017 0.00017     1   85.76  0.0100  0.920775    
Typeofexpression:Group       0.04563 0.01521     3   35.21  0.8870  0.457308    
Typeofexpression:Modality    0.07327 0.02442     3 2388.64  1.4243  0.233744    
Typeofexpression:Group:Modality 0.29010 0.09670     3 2412.08  5.6396  0.000754 ***
Signif. codes:  0 '***' 0.001 '**' 0.01 '*' 0.05 '.' 0.1 ' ' 1
Multiple Comparisons of Means: Tukey Contrasts
Linear Hypotheses: Young adults
               Estimate Std. Error z value Pr(>|z|)
cul - bio == 0  0.02693    0.03311   0.813    0.845
ins - bio == 0  0.06462    0.03775   1.712    0.312
met - bio == 0 -0.01871    0.04559  -0.410    0.976
ins - cul == 0  0.03769    0.03215   1.172    0.639
met - cul == 0 -0.04564    0.04107  -1.111    0.677
met - ins == 0 -0.08333    0.04490  -1.856    0.242
(Adjusted p values reported -- single-step method)
Linear Hypotheses: Children
                Estimate Std. Error z value Pr(>|z|)   
cul - bio == 0 -0.008846   0.027246  -0.325  0.98790   
ins - bio == 0  0.054461   0.030931   1.761  0.28725   
met - bio == 0 -0.078353   0.037359  -2.097  0.15039   
ins - cul == 0  0.063307   0.026344   2.403  0.07448 . 
met - cul == 0 -0.069507   0.033625  -2.067  0.16045   
met - ins == 0 -0.132814   0.036684  -3.620  0.00147 **
---
Signif. codes:  0 '***' 0.001 '**' 0.01 '*' 0.05 '.' 0.1 ' ' 1
(Adjusted p values reported -- single-step method)
Multiple Comparisons of Means: Tukey Contrasts
Linear Hypotheses: Auditory modality-Indivduals with HFA
               Estimate Std. Error z value Pr(>|z|)
cul - bio == 0 -0.01181    0.03069  -0.385    0.980
ins - bio == 0  0.03308    0.03496   0.946    0.776
met - bio == 0 -0.04628    0.04199  -1.102    0.683
ins - cul == 0  0.04489    0.02966   1.514    0.423
met - cul == 0 -0.03447    0.03754  -0.918    0.791
met - ins == 0 -0.07936    0.04117  -1.928    0.212
(Adjusted p values reported -- single-step method)
Linear Hypotheses: Visual modality-Indivduals with HFA
                Estimate Std. Error z value Pr(>|z|)  
cul - bio == 0  0.004273   0.030703   0.139   0.9990  
ins - bio == 0  0.066746   0.034893   1.913   0.2180  
met - bio == 0 -0.038512   0.041992  -0.917   0.7920  
ins - cul == 0  0.062473   0.029638   2.108   0.1470  
met - cul == 0 -0.042784   0.037562  -1.139   0.6601  
met - ins == 0 -0.105258   0.041139  -2.559   0.0501 .
Signif. codes:  0 '***' 0.001 '**' 0.01 '*' 0.05 '.' 0.1 ' ' 1
(Adjusted p values reported -- single-step method)
Linear Hypotheses: Auditory modality-Typically developing individuals
               Estimate Std. Error z value Pr(>|z|)    
cul - bio == 0  0.05021    0.03186   1.576   0.3864    
ins - bio == 0  0.10905    0.03616   3.016   0.0132 *  
met - bio == 0 -0.05612    0.04378  -1.282   0.5687    
ins - cul == 0  0.05883    0.03072   1.915   0.2168    
met - cul == 0 -0.10633    0.03923  -2.710   0.0333 *  
met - ins == 0 -0.16517    0.04285  -3.854   <0.001 ***
Signif. codes:  0 '***' 0.001 '**' 0.01 '*' 0.05 '.' 0.1 ' ' 1
(Adjusted p values reported -- single-step method)
Linear Hypotheses: Visual modality-Typically developing individuals
               Estimate Std. Error z value Pr(>|z|)
cul - bio == 0 -0.01764    0.03777  -0.467    0.965
ins - bio == 0  0.02247    0.04287   0.524    0.952
met - bio == 0 -0.06667    0.05186  -1.286    0.566
ins - cul == 0  0.04012    0.03646   1.100    0.684
met - cul == 0 -0.04903    0.04648  -1.055    0.712
met - ins == 0 -0.08914    0.05080  -1.755    0.290
(Adjusted p values reported -- single-step method)

Fixed effects: Biological idioms
Analysis of Variance Table of type III  with  Satterthwaite approximation for degrees of freedom
             Sum Sq Mean Sq NumDF  DenDF F.value    Pr(>F)    
Age         0.48168 0.48168     1 66.808 31.4251 4.266e-07 ***
Group       0.16995 0.16995     1 54.867 11.0878  0.001558 ** 
Mod         0.00157 0.00157     1 70.031  0.1024  0.749968    
Age:Group   0.00014 0.00014     1 72.909  0.0091  0.924110    
Group:Mod   0.07928 0.07928     1 69.220  5.1721  0.026056 *  
Signif. codes:  0 '***' 0.001 '**' 0.01 '*' 0.05 '.' 0.1 ' ' 1
Fixed effects: Cultural idioms
Analysis of Variance Table of type III  with  Satterthwaite approximation for degrees of freedom
              Sum Sq  Mean Sq NumDF  DenDF F.value    Pr(>F)    
Age         0.272159 0.272159     1 80.919 15.4293 0.0001793 ***
Group       0.111417 0.111417     1 79.101  6.3165 0.0139937 *  
Mod         0.088939 0.088939     1 62.535  5.0422 0.0282814 *  
Age:Group   0.018276 0.018276     1 81.185  1.0361 0.3117488    
Group:Mod   0.039202 0.039202     1 62.623  2.2224 0.1410376    
Signif. codes:  0 '***' 0.001 '**' 0.01 '*' 0.05 '.' 0.1 ' ' 1
Fixed effects: Instructive expressions
Analysis of Variance Table of type III  with  Satterthwaite approximation for degrees of freedom
             Sum Sq Mean Sq NumDF  DenDF F.value    Pr(>F)    
Age         0.33152 0.33152     1  63.51 17.8475 7.794e-05 ***
Group       0.11488 0.11488     1  57.96  6.1846   0.01579 *  
Mod         0.08175 0.08175     1 417.65  4.4010   0.03652 *  
Age:Group   0.01977 0.01977     1  53.35  1.0644   0.30687    
Group:Mod   0.10950 0.10950     1 418.27  5.8950   0.01560 *  
Signif. codes:  0 '***' 0.001 '**' 0.01 '*' 0.05 '.' 0.1 ' ' 1
Fixed effects: Novel metaphors
Analysis of Variance Table of type III  with  Satterthwaite approximation for degrees of freedom
              Sum Sq  Mean Sq NumDF  DenDF F.value    Pr(>F)    
Age         0.124445 0.124445     1 32.719  7.9942 0.0079453 ** 
Group       0.229595 0.229595     1 62.807 14.7489 0.0002883 ***
Mod         0.000144 0.000144     1 68.500  0.0093 0.9236290    
Age:Group   0.001347 0.001347     1 25.388  0.0865 0.7710458    
Group:Mod   0.005904 0.005904     1 68.285  0.3793 0.5400449    
Signif. codes:  0 '***' 0.001 '**' 0.01 '*' 0.05 '.' 0.1 ' ' 1
 
