# Supplementary material for: Can You Play with Fire and Not Hurt Yourself? A Comparative Study in Figurative Language Comprehension between Individuals with and without Autism Spectrum Disorder
Source: PLoS One. 2016 Dec 30;11(12):e0168571. doi: 10.1371/journal.pone.0168571 (PMC5201294; doi:10.1371/journal.pone.0168571)
Supplement: S3 Appendix — (RTF) [file pone.0168571.s003.rtf]

Appendix S3. Overall model (Accuracy analysis: Target vs non-target)
Generalized linear mixed model fit by maximum likelihood (Laplace approximation) ['glmerMod']
Formula: error ~ Age2 * Group2 + Mod2 + Typeofexpression + (1 + Typeofexpression |part) + (1 + Age2 + Group2 | condition)
Control: glmerControl(optimizer = "bobyqa", optCtrl = list(maxfun = 1e+05))
Fixed effects:                
 Df   Chisq Chi Df Pr(>Chisq)    
Age              23  5.7376      1  0.0166058 *  
Group            23 11.2104      1  0.0008134 ***
Mod              23  0.1678      1  0.6821015    
Typeofexpression 21  8.4945      3  0.0368243 *  
Age:Group        23  4.9872      1  0.0255350 *  
Signif. codes:  0 '***' 0.001 '**' 0.01 '*' 0.05 '.' 0.1 ' ' 1
Multiple Comparisons of Means: Tukey Contrasts
Linear Hypotheses:
                               Estimate  Std. Error  z value   Pr(>|z|)    
Cont adul- ASD adul == 0        -1.4608     0.3805  -3.839   <0.001 ***
ASD chil – ASD adul == 0         0.1025     0.3396   0.302   0.9904    
Cont chil – ASD adul == 0       -0.2392     0.3652  -0.655   0.9134    
ASD chil – Cont adul == 0       1.5633     0.3941   3.967   <0.001 ***
Cont chil – Control adul == 0   1.2216     0.3934   3.105   0.0104 *  
Cont chil – ASD chil == 0      -0.3417     0.3414  -1.001   0.7477    
Signif. codes:  0 '***' 0.001 '**' 0.01 '*' 0.05 '.' 0.1 ' ' 1
(Adjusted p values reported -- single-step method)
Linear Hypotheses:
               Estimate Std. Error z value Pr(>|z|)  
cul - bio == 0   0.5014     0.2987   1.679   0.3265  
ins - bio == 0   0.8149     0.3366   2.421   0.0700 .
met - bio == 0  -0.1513     0.4008  -0.378   0.9810  
ins - cul == 0   0.3135     0.2492   1.258   0.5812  
met - cul == 0  -0.6527     0.3623  -1.802   0.2648  
met - ins == 0  -0.9662     0.3965  -2.437   0.0672 .
Signif. codes:  0 '***' 0.001 '**' 0.01 '*' 0.05 '.' 0.1 ' ' 1
(Adjusted p values reported -- single-step method)
