# Supplementary material for: Can You Play with Fire and Not Hurt Yourself? A Comparative Study in Figurative Language Comprehension between Individuals with and without Autism Spectrum Disorder
Source: PLoS One. 2016 Dec 30;11(12):e0168571. doi: 10.1371/journal.pone.0168571 (PMC5201294; doi:10.1371/journal.pone.0168571)
Supplement: S4 Appendix — (RTF) [file pone.0168571.s004.rtf]

Appendix S4. Overall model (Accuracy analysis: Target vs literal responses)
Generalized linear mixed model fit by maximum likelihood (Laplace Approximation) ['glmerMod']
Formula: AvsBrec ~ Age2 * Group2 * Mod2 + Typeofexpression + (1 + Typeofexpression |part) + (1 + Age2 + Group2 | condition)
Fixed effects:
                 Df   Chisq Chi Df Pr(>Chisq)  
Age              26  3.5160      1    0.06078 .
Group            26  5.2293      1    0.02221 *
Mod              26  1.5193      1    0.21772  
Typeofexpression 24 10.3722      3    0.01565 *
Age:Group        26  4.8956      1    0.02692 *
Age:Mod          26  0.1523      1    0.69638  
Group:Mod        26  1.2132      1    0.27069  
Age:Group:Mod    26  3.9749      1    0.04618 *
Signif. codes:  0 '***' 0.001 '**' 0.01 '*' 0.05 '.' 0.1 ' ' 1
         Simultaneous Tests for General Linear Hypotheses


Multiple Comparisons of Means: Tukey Contrasts
Linear Hypotheses:
                               Estimate Std. Error z value Pr(>|z|)  
Cont adul- ASD adul == 0       -1.68456    0.56811  -2.965   0.0158 *
ASD chil – ASD adul == 0       -0.03629    0.49160  -0.074   0.9999  
Cont chil – ASD adul == 0      -0.13918    0.52898  -0.263   0.9936  
ASD chil – Cont adul == 0       1.64827    0.59157   2.786   0.0270 *
Cont chil – Control adul == 0   1.54537    0.57966   2.666   0.0379 *
Cont chil – ASD chil == 0      -0.10289    0.48884  -0.210   0.9967  
Signif. codes:  0 '***' 0.001 '**' 0.01 '*' 0.05 '.' 0.1 ' ' 1
(Adjusted p values reported -- single-step method)
Linear Hypotheses:
               Estimate Std. Error z value Pr(>|z|)  
cul - bio == 0   0.1785     0.4229   0.422   0.9727  
ins - bio == 0   0.6033     0.4767   1.266   0.5655  
met - bio == 0  -1.7250     0.8418  -2.049   0.1569  
ins - cul == 0   0.4247     0.3586   1.184   0.6184  
met - cul == 0  -1.9035     0.8150  -2.336   0.0818 .
met - ins == 0  -2.3283     0.8564  -2.719   0.0297 *
Signif. codes:  0 '***' 0.001 '**' 0.01 '*' 0.05 '.' 0.1 ' ' 1
(Adjusted p values reported -- single-step method)
Linear Hypotheses: Visual modality
                               Estimate Std. Error z value Pr(>|z|)
Cont adul- ASD adul == 0       -0.94103    0.69354  -1.357    0.525
ASD chil – ASD adul == 0        0.19587    0.65685   0.298    0.991
Cont chil – ASD adul == 0       0.14371    0.71234   0.202    0.997
ASD chil – Cont adul == 0       1.13690    0.73950   1.537    0.414
Cont chil – Control adul == 0   1.08473    0.73594   1.474    0.452
Cont chil – ASD chil == 0      -0.05217    0.65204  -0.080    1.000
(Adjusted p values reported -- single-step method)
Linear Hypotheses: Auditory modality
                               Estimate Std. Error z value Pr(>|z|)   
Cont adul- ASD adul == 0        -2.8033     0.7610  -3.684   0.0014 **
ASD chil – ASD adul == 0        -0.4233     0.5383  -0.786   0.8580  
Cont chil – ASD adul == 0       -0.9072     0.6437  -1.409   0.4877   
ASD chil – Cont adul == 0        2.3800     0.7766   3.065   0.0118 * 
Cont chil – Control adul == 0    1.8962     0.7529   2.518   0.0555 . 
Cont chil – ASD chil == 0       -0.4838     0.5987  -0.808   0.8478   
Signif. codes:  0 '***' 0.001 '**' 0.01 '*' 0.05 '.' 0.1 ' ' 1
(Adjusted p values reported -- single-step method)
