# Supplementary material for: Can You Play with Fire and Not Hurt Yourself? A Comparative Study in Figurative Language Comprehension between Individuals with and without Autism Spectrum Disorder
Source: PLoS One. 2016 Dec 30;11(12):e0168571. doi: 10.1371/journal.pone.0168571 (PMC5201294; doi:10.1371/journal.pone.0168571)
Supplement: S5 Appendix — (RTF) [file pone.0168571.s005.rtf]

Appendix S5. Overall model (Accuracy analysis: Figurative target vs Figurative non-target responses)
Generalized linear mixed model fit by maximum likelihood (Laplace Approximation) ['glmerMod']
 Formula: AvsCrec ~ Age2 * Group2 + Mod2 + Typeofexpression + (1 | part) +      (1 + Age2 | condition)
Fixed effects:
                 Df   Chisq Chi Df Pr(>Chisq)    
Age              11 12.9216      1  0.0003248 ***
Group            11  6.4983      1  0.0107980 *  
Mod              11  0.1722      1  0.6781582    
Typeofexpression  9  5.1595      3  0.1604857    
Age:Group        11  2.4667      1  0.1162855    
Signif. codes:  0 '***' 0.001 '**' 0.01 '*' 0.05 '.' 0.1 ' ' 1
