# Supplementary material for: Can You Play with Fire and Not Hurt Yourself? A Comparative Study in Figurative Language Comprehension between Individuals with and without Autism Spectrum Disorder
Source: PLoS One. 2016 Dec 30;11(12):e0168571. doi: 10.1371/journal.pone.0168571 (PMC5201294; doi:10.1371/journal.pone.0168571)
Supplement: S6 Appendix — (DOCX) [file pone.0168571.s006.docx]

**Appendix S6. General linear model analyses with receptive grammar as covariate**

*Reaction time*

| **Multivariate Tests** | | | | | | |
| --- | --- | --- | --- | --- | --- | --- |
| Effect | | Value | F | Hypothesis df | Error df | Sig. |
| mod | Pillai's Trace | ,004 | ,262^b^ | 1,000 | 74,000 | ,610 |
|  | Wilks' Lambda | ,996 | ,262^b^ | 1,000 | 74,000 | ,610 |
|  | Hotelling's Trace | ,004 | ,262^b^ | 1,000 | 74,000 | ,610 |
|  | Roy's Largest Root | ,004 | ,262^b^ | 1,000 | 74,000 | ,610 |
| mod * CEGscorebyitem_mean | Pillai's Trace | ,003 | ,192^b^ | 1,000 | 74,000 | ,662 |
|  | Wilks' Lambda | ,997 | ,192^b^ | 1,000 | 74,000 | ,662 |
|  | Hotelling's Trace | ,003 | ,192^b^ | 1,000 | 74,000 | ,662 |
|  | Roy's Largest Root | ,003 | ,192^b^ | 1,000 | 74,000 | ,662 |
| mod * AgexGroup | Pillai's Trace | ,026 | ,664^b^ | 3,000 | 74,000 | ,577 |
|  | Wilks' Lambda | ,974 | ,664^b^ | 3,000 | 74,000 | ,577 |
|  | Hotelling's Trace | ,027 | ,664^b^ | 3,000 | 74,000 | ,577 |
|  | Roy's Largest Root | ,027 | ,664^b^ | 3,000 | 74,000 | ,577 |
| typeofexpression | Pillai's Trace | ,031 | ,760^b^ | 3,000 | 72,000 | ,520 |
|  | Wilks' Lambda | ,969 | ,760^b^ | 3,000 | 72,000 | ,520 |
|  | Hotelling's Trace | ,032 | ,760^b^ | 3,000 | 72,000 | ,520 |
|  | Roy's Largest Root | ,032 | ,760^b^ | 3,000 | 72,000 | ,520 |
| typeofexpression * CEGscorebyitem_mean | Pillai's Trace | ,043 | 1,080^b^ | 3,000 | 72,000 | ,363 |
|  | Wilks' Lambda | ,957 | 1,080^b^ | 3,000 | 72,000 | ,363 |
|  | Hotelling's Trace | ,045 | 1,080^b^ | 3,000 | 72,000 | ,363 |
|  | Roy's Largest Root | ,045 | 1,080^b^ | 3,000 | 72,000 | ,363 |
| typeofexpression * AgexGroup | Pillai's Trace | ,172 | 1,498 | 9,000 | 222,000 | ,150 |
|  | Wilks' Lambda | ,835 | 1,497 | 9,000 | 175,380 | ,152 |
|  | Hotelling's Trace | ,189 | 1,485 | 9,000 | 212,000 | ,155 |
|  | Roy's Largest Root | ,123 | 3,026^c^ | 3,000 | 74,000 | ,035 |
| mod * typeofexpression | Pillai's Trace | ,049 | 1,236^b^ | 3,000 | 72,000 | ,303 |
|  | Wilks' Lambda | ,951 | 1,236^b^ | 3,000 | 72,000 | ,303 |
|  | Hotelling's Trace | ,052 | 1,236^b^ | 3,000 | 72,000 | ,303 |
|  | Roy's Largest Root | ,052 | 1,236^b^ | 3,000 | 72,000 | ,303 |
| mod * typeofexpression * CEGscorebyitem_mean | Pillai's Trace | ,051 | 1,284^b^ | 3,000 | 72,000 | ,286 |
|  | Wilks' Lambda | ,949 | 1,284^b^ | 3,000 | 72,000 | ,286 |
|  | Hotelling's Trace | ,054 | 1,284^b^ | 3,000 | 72,000 | ,286 |
|  | Roy's Largest Root | ,054 | 1,284^b^ | 3,000 | 72,000 | ,286 |
| mod * typeofexpression * AgexGroup | Pillai's Trace | ,122 | 1,044 | 9,000 | 222,000 | ,406 |
|  | Wilks' Lambda | ,880 | 1,050 | 9,000 | 175,380 | ,402 |
|  | Hotelling's Trace | ,134 | 1,054 | 9,000 | 212,000 | ,399 |
|  | Roy's Largest Root | ,117 | 2,885^c^ | 3,000 | 74,000 | ,041 |

| **Tests of Within-Subjects Effects** | | | | | | | |
| --- | --- | --- | --- | --- | --- | --- | --- |
|  | | | | | | | |
| Source | | Type III Sum of Squares | df | Mean Square | F | Sig. | Partial Eta Squared |
| mod | Sphericity Assumed | 1673090,706 | 1 | 1673090,706 | ,262 | ,610 | ,004 |
|  | Greenhouse-Geisser | 1673090,706 | 1,000 | 1673090,706 | ,262 | ,610 | ,004 |
|  | Huynh-Feldt | 1673090,706 | 1,000 | 1673090,706 | ,262 | ,610 | ,004 |
|  | Lower-bound | 1673090,706 | 1,000 | 1673090,706 | ,262 | ,610 | ,004 |
| mod * CEGscorebyitem_mean | Sphericity Assumed | 1229788,645 | 1 | 1229788,645 | ,192 | ,662 | ,003 |
|  | Greenhouse-Geisser | 1229788,645 | 1,000 | 1229788,645 | ,192 | ,662 | ,003 |
|  | Huynh-Feldt | 1229788,645 | 1,000 | 1229788,645 | ,192 | ,662 | ,003 |
|  | Lower-bound | 1229788,645 | 1,000 | 1229788,645 | ,192 | ,662 | ,003 |
| mod * AgexGroup | Sphericity Assumed | 12727211,645 | 3 | 4242403,882 | ,664 | ,577 | ,026 |
|  | Greenhouse-Geisser | 12727211,645 | 3,000 | 4242403,882 | ,664 | ,577 | ,026 |
|  | Huynh-Feldt | 12727211,645 | 3,000 | 4242403,882 | ,664 | ,577 | ,026 |
|  | Lower-bound | 12727211,645 | 3,000 | 4242403,882 | ,664 | ,577 | ,026 |
| Error(mod) | Sphericity Assumed | 473086862,454 | 74 | 6393065,709 |  |  |  |
|  | Greenhouse-Geisser | 473086862,454 | 74,000 | 6393065,709 |  |  |  |
|  | Huynh-Feldt | 473086862,454 | 74,000 | 6393065,709 |  |  |  |
|  | Lower-bound | 473086862,454 | 74,000 | 6393065,709 |  |  |  |
| typeofexpression | Sphericity Assumed | 6952405,190 | 3 | 2317468,397 | ,326 | ,807 | ,004 |
|  | Greenhouse-Geisser | 6952405,190 | 1,945 | 3573604,433 | ,326 | ,716 | ,004 |
|  | Huynh-Feldt | 6952405,190 | 2,105 | 3302395,573 | ,326 | ,733 | ,004 |
|  | Lower-bound | 6952405,190 | 1,000 | 6952405,190 | ,326 | ,570 | ,004 |
| typeofexpression * CEGscorebyitem_mean | Sphericity Assumed | 12091136,113 | 3 | 4030378,704 | ,567 | ,637 | ,008 |
|  | Greenhouse-Geisser | 12091136,113 | 1,945 | 6214962,510 | ,567 | ,564 | ,008 |
|  | Huynh-Feldt | 12091136,113 | 2,105 | 5743295,058 | ,567 | ,577 | ,008 |
|  | Lower-bound | 12091136,113 | 1,000 | 12091136,113 | ,567 | ,454 | ,008 |
| typeofexpression * AgexGroup | Sphericity Assumed | 79150644,826 | 9 | 8794516,092 | 1,237 | ,273 | ,048 |
|  | Greenhouse-Geisser | 79150644,826 | 5,836 | 13561402,492 | 1,237 | ,291 | ,048 |
|  | Huynh-Feldt | 79150644,826 | 6,316 | 12532197,224 | 1,237 | ,289 | ,048 |
|  | Lower-bound | 79150644,826 | 3,000 | 26383548,275 | 1,237 | ,302 | ,048 |
| Error(typeofexpression) | Sphericity Assumed | 1578151527,396 | 222 | 7108790,664 |  |  |  |
|  | Greenhouse-Geisser | 1578151527,396 | 143,966 | 10961964,299 |  |  |  |
|  | Huynh-Feldt | 1578151527,396 | 155,789 | 10130036,228 |  |  |  |
|  | Lower-bound | 1578151527,396 | 74,000 | 21326371,992 |  |  |  |
| mod * typeofexpression | Sphericity Assumed | 18287392,167 | 3 | 6095797,389 | ,882 | ,451 | ,012 |
|  | Greenhouse-Geisser | 18287392,167 | 1,477 | 12378809,542 | ,882 | ,389 | ,012 |
|  | Huynh-Feldt | 18287392,167 | 1,582 | 11561990,248 | ,882 | ,395 | ,012 |
|  | Lower-bound | 18287392,167 | 1,000 | 18287392,167 | ,882 | ,351 | ,012 |
| mod * typeofexpression * CEGscorebyitem_mean | Sphericity Assumed | 20259358,756 | 3 | 6753119,585 | ,977 | ,405 | ,013 |
|  | Greenhouse-Geisser | 20259358,756 | 1,477 | 13713641,682 | ,977 | ,357 | ,013 |
|  | Huynh-Feldt | 20259358,756 | 1,582 | 12808743,107 | ,977 | ,362 | ,013 |
|  | Lower-bound | 20259358,756 | 1,000 | 20259358,756 | ,977 | ,326 | ,013 |
| mod * typeofexpression * AgexGroup | Sphericity Assumed | 41171563,287 | 9 | 4574618,143 | ,662 | ,743 | ,026 |
|  | Greenhouse-Geisser | 41171563,287 | 4,432 | 9289732,434 | ,662 | ,635 | ,026 |
|  | Huynh-Feldt | 41171563,287 | 4,745 | 8676746,779 | ,662 | ,645 | ,026 |
|  | Lower-bound | 41171563,287 | 3,000 | 13723854,429 | ,662 | ,578 | ,026 |
| Error(mod*typeofexpression) | Sphericity Assumed | 1535078032,873 | 222 | 6914765,914 |  |  |  |
|  | Greenhouse-Geisser | 1535078032,873 | 109,321 | 14041898,838 |  |  |  |
|  | Huynh-Feldt | 1535078032,873 | 117,044 | 13115340,121 |  |  |  |
|  | Lower-bound | 1535078032,873 | 74,000 | 20744297,742 |  |  |  |

*Overall accuracy*

| **Multivariate Tests** | | | | | | |
| --- | --- | --- | --- | --- | --- | --- |
| Effect | | Value | F | Hypothesis df | Error df | Sig. |
| mod | Pillai's Trace | ,007 | ,538^b^ | 1,000 | 74,000 | ,466 |
|  | Wilks' Lambda | ,993 | ,538^b^ | 1,000 | 74,000 | ,466 |
|  | Hotelling's Trace | ,007 | ,538^b^ | 1,000 | 74,000 | ,466 |
|  | Roy's Largest Root | ,007 | ,538^b^ | 1,000 | 74,000 | ,466 |
| mod * CEGscorebyitem_mean | Pillai's Trace | ,008 | ,586^b^ | 1,000 | 74,000 | ,447 |
|  | Wilks' Lambda | ,992 | ,586^b^ | 1,000 | 74,000 | ,447 |
|  | Hotelling's Trace | ,008 | ,586^b^ | 1,000 | 74,000 | ,447 |
|  | Roy's Largest Root | ,008 | ,586^b^ | 1,000 | 74,000 | ,447 |
| mod * AgexGroup | Pillai's Trace | ,039 | ,994^b^ | 3,000 | 74,000 | ,400 |
|  | Wilks' Lambda | ,961 | ,994^b^ | 3,000 | 74,000 | ,400 |
|  | Hotelling's Trace | ,040 | ,994^b^ | 3,000 | 74,000 | ,400 |
|  | Roy's Largest Root | ,040 | ,994^b^ | 3,000 | 74,000 | ,400 |
| typeofexpression | Pillai's Trace | ,016 | ,382^b^ | 3,000 | 72,000 | ,766 |
|  | Wilks' Lambda | ,984 | ,382^b^ | 3,000 | 72,000 | ,766 |
|  | Hotelling's Trace | ,016 | ,382^b^ | 3,000 | 72,000 | ,766 |
|  | Roy's Largest Root | ,016 | ,382^b^ | 3,000 | 72,000 | ,766 |
| typeofexpression * CEGscorebyitem_mean | Pillai's Trace | ,013 | ,317^b^ | 3,000 | 72,000 | ,813 |
|  | Wilks' Lambda | ,987 | ,317^b^ | 3,000 | 72,000 | ,813 |
|  | Hotelling's Trace | ,013 | ,317^b^ | 3,000 | 72,000 | ,813 |
|  | Roy's Largest Root | ,013 | ,317^b^ | 3,000 | 72,000 | ,813 |
| typeofexpression * AgexGroup | Pillai's Trace | ,080 | ,672 | 9,000 | 222,000 | ,734 |
|  | Wilks' Lambda | ,922 | ,662 | 9,000 | 175,380 | ,742 |
|  | Hotelling's Trace | ,083 | ,653 | 9,000 | 212,000 | ,750 |
|  | Roy's Largest Root | ,056 | 1,381^c^ | 3,000 | 74,000 | ,255 |
| mod * typeofexpression | Pillai's Trace | ,005 | ,117^b^ | 3,000 | 72,000 | ,950 |
|  | Wilks' Lambda | ,995 | ,117^b^ | 3,000 | 72,000 | ,950 |
|  | Hotelling's Trace | ,005 | ,117^b^ | 3,000 | 72,000 | ,950 |
|  | Roy's Largest Root | ,005 | ,117^b^ | 3,000 | 72,000 | ,950 |
| mod * typeofexpression * CEGscorebyitem_mean | Pillai's Trace | ,004 | ,085^b^ | 3,000 | 72,000 | ,968 |
|  | Wilks' Lambda | ,996 | ,085^b^ | 3,000 | 72,000 | ,968 |
|  | Hotelling's Trace | ,004 | ,085^b^ | 3,000 | 72,000 | ,968 |
|  | Roy's Largest Root | ,004 | ,085^b^ | 3,000 | 72,000 | ,968 |
| mod * typeofexpression * AgexGroup | Pillai's Trace | ,136 | 1,172 | 9,000 | 222,000 | ,314 |
|  | Wilks' Lambda | ,866 | 1,182 | 9,000 | 175,380 | ,309 |
|  | Hotelling's Trace | ,151 | 1,187 | 9,000 | 212,000 | ,304 |
|  | Roy's Largest Root | ,129 | 3,179^c^ | 3,000 | 74,000 | ,029 |

| **Tests of Within-Subjects Effects** | | | | | | | |
| --- | --- | --- | --- | --- | --- | --- | --- |
|  | | | | | | | |
| Source | | Type III Sum of Squares | df | Mean Square | F | Sig. | Partial Eta Squared |
| mod | Sphericity Assumed | ,020 | 1 | ,020 | ,538 | ,466 | ,007 |
|  | Greenhouse-Geisser | ,020 | 1,000 | ,020 | ,538 | ,466 | ,007 |
|  | Huynh-Feldt | ,020 | 1,000 | ,020 | ,538 | ,466 | ,007 |
|  | Lower-bound | ,020 | 1,000 | ,020 | ,538 | ,466 | ,007 |
| mod * CEGscorebyitem_mean | Sphericity Assumed | ,021 | 1 | ,021 | ,586 | ,447 | ,008 |
|  | Greenhouse-Geisser | ,021 | 1,000 | ,021 | ,586 | ,447 | ,008 |
|  | Huynh-Feldt | ,021 | 1,000 | ,021 | ,586 | ,447 | ,008 |
|  | Lower-bound | ,021 | 1,000 | ,021 | ,586 | ,447 | ,008 |
| mod * AgexGroup | Sphericity Assumed | ,109 | 3 | ,036 | ,994 | ,400 | ,039 |
|  | Greenhouse-Geisser | ,109 | 3,000 | ,036 | ,994 | ,400 | ,039 |
|  | Huynh-Feldt | ,109 | 3,000 | ,036 | ,994 | ,400 | ,039 |
|  | Lower-bound | ,109 | 3,000 | ,036 | ,994 | ,400 | ,039 |
| Error(mod) | Sphericity Assumed | 2,695 | 74 | ,036 |  |  |  |
|  | Greenhouse-Geisser | 2,695 | 74,000 | ,036 |  |  |  |
|  | Huynh-Feldt | 2,695 | 74,000 | ,036 |  |  |  |
|  | Lower-bound | 2,695 | 74,000 | ,036 |  |  |  |
| typeofexpression | Sphericity Assumed | ,025 | 3 | ,008 | ,295 | ,829 | ,004 |
|  | Greenhouse-Geisser | ,025 | 2,325 | ,011 | ,295 | ,778 | ,004 |
|  | Huynh-Feldt | ,025 | 2,535 | ,010 | ,295 | ,796 | ,004 |
|  | Lower-bound | ,025 | 1,000 | ,025 | ,295 | ,589 | ,004 |
| typeofexpression * CEGscorebyitem_mean | Sphericity Assumed | ,026 | 3 | ,009 | ,300 | ,825 | ,004 |
|  | Greenhouse-Geisser | ,026 | 2,325 | ,011 | ,300 | ,774 | ,004 |
|  | Huynh-Feldt | ,026 | 2,535 | ,010 | ,300 | ,792 | ,004 |
|  | Lower-bound | ,026 | 1,000 | ,026 | ,300 | ,585 | ,004 |
| typeofexpression * AgexGroup | Sphericity Assumed | ,147 | 9 | ,016 | ,575 | ,817 | ,023 |
|  | Greenhouse-Geisser | ,147 | 6,976 | ,021 | ,575 | ,775 | ,023 |
|  | Huynh-Feldt | ,147 | 7,606 | ,019 | ,575 | ,789 | ,023 |
|  | Lower-bound | ,147 | 3,000 | ,049 | ,575 | ,633 | ,023 |
| Error(typeofexpression) | Sphericity Assumed | 6,316 | 222 | ,028 |  |  |  |
|  | Greenhouse-Geisser | 6,316 | 172,083 | ,037 |  |  |  |
|  | Huynh-Feldt | 6,316 | 187,605 | ,034 |  |  |  |
|  | Lower-bound | 6,316 | 74,000 | ,085 |  |  |  |
| mod * typeofexpression | Sphericity Assumed | ,011 | 3 | ,004 | ,133 | ,940 | ,002 |
|  | Greenhouse-Geisser | ,011 | 2,566 | ,004 | ,133 | ,919 | ,002 |
|  | Huynh-Feldt | ,011 | 2,810 | ,004 | ,133 | ,932 | ,002 |
|  | Lower-bound | ,011 | 1,000 | ,011 | ,133 | ,716 | ,002 |
| mod * typeofexpression * CEGscorebyitem_mean | Sphericity Assumed | ,008 | 3 | ,003 | ,092 | ,964 | ,001 |
|  | Greenhouse-Geisser | ,008 | 2,566 | ,003 | ,092 | ,948 | ,001 |
|  | Huynh-Feldt | ,008 | 2,810 | ,003 | ,092 | ,958 | ,001 |
|  | Lower-bound | ,008 | 1,000 | ,008 | ,092 | ,762 | ,001 |
| mod * typeofexpression * AgexGroup | Sphericity Assumed | ,403 | 9 | ,045 | 1,633 | ,107 | ,062 |
|  | Greenhouse-Geisser | ,403 | 7,698 | ,052 | 1,633 | ,121 | ,062 |
|  | Huynh-Feldt | ,403 | 8,429 | ,048 | 1,633 | ,113 | ,062 |
|  | Lower-bound | ,403 | 3,000 | ,134 | 1,633 | ,189 | ,062 |
| Error(mod*typeofexpression) | Sphericity Assumed | 6,091 | 222 | ,027 |  |  |  |
|  | Greenhouse-Geisser | 6,091 | 189,879 | ,032 |  |  |  |
|  | Huynh-Feldt | 6,091 | 207,918 | ,029 |  |  |  |
|  | Lower-bound | 6,091 | 74,000 | ,082 |  |  |  |

*Comparison between target and literal responses*

| **Multivariate Tests** | | | | | | |
| --- | --- | --- | --- | --- | --- | --- |
| Effect | | Value | F | Hypothesis df | Error df | Sig. |
| mod | Pillai's Trace | ,013 | ,956^b^ | 1,000 | 74,000 | ,331 |
|  | Wilks' Lambda | ,987 | ,956^b^ | 1,000 | 74,000 | ,331 |
|  | Hotelling's Trace | ,013 | ,956^b^ | 1,000 | 74,000 | ,331 |
|  | Roy's Largest Root | ,013 | ,956^b^ | 1,000 | 74,000 | ,331 |
| mod * CEGscorebyitem_mean | Pillai's Trace | ,013 | ,953^b^ | 1,000 | 74,000 | ,332 |
|  | Wilks' Lambda | ,987 | ,953^b^ | 1,000 | 74,000 | ,332 |
|  | Hotelling's Trace | ,013 | ,953^b^ | 1,000 | 74,000 | ,332 |
|  | Roy's Largest Root | ,013 | ,953^b^ | 1,000 | 74,000 | ,332 |
| mod * AgexGroup | Pillai's Trace | ,021 | ,519^b^ | 3,000 | 74,000 | ,671 |
|  | Wilks' Lambda | ,979 | ,519^b^ | 3,000 | 74,000 | ,671 |
|  | Hotelling's Trace | ,021 | ,519^b^ | 3,000 | 74,000 | ,671 |
|  | Roy's Largest Root | ,021 | ,519^b^ | 3,000 | 74,000 | ,671 |
| typeofexpression | Pillai's Trace | ,031 | ,780^b^ | 3,000 | 72,000 | ,509 |
|  | Wilks' Lambda | ,969 | ,780^b^ | 3,000 | 72,000 | ,509 |
|  | Hotelling's Trace | ,033 | ,780^b^ | 3,000 | 72,000 | ,509 |
|  | Roy's Largest Root | ,033 | ,780^b^ | 3,000 | 72,000 | ,509 |
| typeofexpression * CEGscorebyitem_mean | Pillai's Trace | ,025 | ,617^b^ | 3,000 | 72,000 | ,606 |
|  | Wilks' Lambda | ,975 | ,617^b^ | 3,000 | 72,000 | ,606 |
|  | Hotelling's Trace | ,026 | ,617^b^ | 3,000 | 72,000 | ,606 |
|  | Roy's Largest Root | ,026 | ,617^b^ | 3,000 | 72,000 | ,606 |
| typeofexpression * AgexGroup | Pillai's Trace | ,048 | ,398 | 9,000 | 222,000 | ,935 |
|  | Wilks' Lambda | ,953 | ,389 | 9,000 | 175,380 | ,939 |
|  | Hotelling's Trace | ,049 | ,382 | 9,000 | 212,000 | ,943 |
|  | Roy's Largest Root | ,029 | ,725^c^ | 3,000 | 74,000 | ,540 |
| mod * typeofexpression | Pillai's Trace | ,008 | ,190^b^ | 3,000 | 72,000 | ,903 |
|  | Wilks' Lambda | ,992 | ,190^b^ | 3,000 | 72,000 | ,903 |
|  | Hotelling's Trace | ,008 | ,190^b^ | 3,000 | 72,000 | ,903 |
|  | Roy's Largest Root | ,008 | ,190^b^ | 3,000 | 72,000 | ,903 |
| mod * typeofexpression * CEGscorebyitem_mean | Pillai's Trace | ,007 | ,161^b^ | 3,000 | 72,000 | ,922 |
|  | Wilks' Lambda | ,993 | ,161^b^ | 3,000 | 72,000 | ,922 |
|  | Hotelling's Trace | ,007 | ,161^b^ | 3,000 | 72,000 | ,922 |
|  | Roy's Largest Root | ,007 | ,161^b^ | 3,000 | 72,000 | ,922 |
| mod * typeofexpression * AgexGroup | Pillai's Trace | ,148 | 1,280 | 9,000 | 222,000 | ,249 |
|  | Wilks' Lambda | ,858 | 1,270 | 9,000 | 175,380 | ,256 |
|  | Hotelling's Trace | ,160 | 1,253 | 9,000 | 212,000 | ,264 |
|  | Roy's Largest Root | ,106 | 2,604^c^ | 3,000 | 74,000 | ,058 |

| **Tests of Within-Subjects Effects** | | | |
| --- | --- | --- | --- |
|  | | | |
| Source | | Noncent. Parameter | Observed Power^a^ |
| mod | Sphericity Assumed | ,956 | ,162 |
|  | Greenhouse-Geisser | ,956 | ,162 |
|  | Huynh-Feldt | ,956 | ,162 |
|  | Lower-bound | ,956 | ,162 |
| mod * CEGscorebyitem_mean | Sphericity Assumed | ,953 | ,161 |
|  | Greenhouse-Geisser | ,953 | ,161 |
|  | Huynh-Feldt | ,953 | ,161 |
|  | Lower-bound | ,953 | ,161 |
| mod * AgexGroup | Sphericity Assumed | 1,556 | ,151 |
|  | Greenhouse-Geisser | 1,556 | ,151 |
|  | Huynh-Feldt | 1,556 | ,151 |
|  | Lower-bound | 1,556 | ,151 |
| Error(mod) | Sphericity Assumed |  |  |
|  | Greenhouse-Geisser |  |  |
|  | Huynh-Feldt |  |  |
|  | Lower-bound |  |  |
| typeofexpression | Sphericity Assumed | 3,200 | ,287 |
|  | Greenhouse-Geisser | 2,378 | ,247 |
|  | Huynh-Feldt | 2,588 | ,257 |
|  | Lower-bound | 1,067 | ,175 |
| typeofexpression * CEGscorebyitem_mean | Sphericity Assumed | 2,742 | ,249 |
|  | Greenhouse-Geisser | 2,038 | ,216 |
|  | Huynh-Feldt | 2,218 | ,225 |
|  | Lower-bound | ,914 | ,157 |
| typeofexpression * AgexGroup | Sphericity Assumed | 3,544 | ,194 |
|  | Greenhouse-Geisser | 2,634 | ,169 |
|  | Huynh-Feldt | 2,867 | ,176 |
|  | Lower-bound | 1,181 | ,124 |
| Error(typeofexpression) | Sphericity Assumed |  |  |
|  | Greenhouse-Geisser |  |  |
|  | Huynh-Feldt |  |  |
|  | Lower-bound |  |  |
| mod * typeofexpression | Sphericity Assumed | ,843 | ,103 |
|  | Greenhouse-Geisser | ,634 | ,096 |
|  | Huynh-Feldt | ,691 | ,098 |
|  | Lower-bound | ,281 | ,082 |
| mod * typeofexpression * CEGscorebyitem_mean | Sphericity Assumed | ,703 | ,094 |
|  | Greenhouse-Geisser | ,529 | ,088 |
|  | Huynh-Feldt | ,577 | ,090 |
|  | Lower-bound | ,234 | ,077 |
| mod * typeofexpression * AgexGroup | Sphericity Assumed | 13,554 | ,707 |
|  | Greenhouse-Geisser | 10,204 | ,610 |
|  | Huynh-Feldt | 11,110 | ,638 |
|  | Lower-bound | 4,518 | ,382 |
| Error(mod*typeofexpression) | Sphericity Assumed |  |  |
|  | Greenhouse-Geisser |  |  |
|  | Huynh-Feldt |  |  |
|  | Lower-bound |  |  |

*Comparison between target and figurative non target responses*

| **Multivariate Tests** | | | | | | |
| --- | --- | --- | --- | --- | --- | --- |
| Effect | | Value | F | Hypothesis df | Error df | Sig. |
| mod | Pillai's Trace | ,011 | ,771^b^ | 1,000 | 69,000 | ,383 |
|  | Wilks' Lambda | ,989 | ,771^b^ | 1,000 | 69,000 | ,383 |
|  | Hotelling's Trace | ,011 | ,771^b^ | 1,000 | 69,000 | ,383 |
|  | Roy's Largest Root | ,011 | ,771^b^ | 1,000 | 69,000 | ,383 |
| mod * CEGscorebyitem_mean | Pillai's Trace | ,011 | ,794^b^ | 1,000 | 69,000 | ,376 |
|  | Wilks' Lambda | ,989 | ,794^b^ | 1,000 | 69,000 | ,376 |
|  | Hotelling's Trace | ,012 | ,794^b^ | 1,000 | 69,000 | ,376 |
|  | Roy's Largest Root | ,012 | ,794^b^ | 1,000 | 69,000 | ,376 |
| mod * AgexGroup | Pillai's Trace | ,011 | ,250^b^ | 3,000 | 69,000 | ,861 |
|  | Wilks' Lambda | ,989 | ,250^b^ | 3,000 | 69,000 | ,861 |
|  | Hotelling's Trace | ,011 | ,250^b^ | 3,000 | 69,000 | ,861 |
|  | Roy's Largest Root | ,011 | ,250^b^ | 3,000 | 69,000 | ,861 |
| typeofexpression | Pillai's Trace | ,073 | 1,750^b^ | 3,000 | 67,000 | ,165 |
|  | Wilks' Lambda | ,927 | 1,750^b^ | 3,000 | 67,000 | ,165 |
|  | Hotelling's Trace | ,078 | 1,750^b^ | 3,000 | 67,000 | ,165 |
|  | Roy's Largest Root | ,078 | 1,750^b^ | 3,000 | 67,000 | ,165 |
| typeofexpression * CEGscorebyitem_mean | Pillai's Trace | ,062 | 1,478^b^ | 3,000 | 67,000 | ,228 |
|  | Wilks' Lambda | ,938 | 1,478^b^ | 3,000 | 67,000 | ,228 |
|  | Hotelling's Trace | ,066 | 1,478^b^ | 3,000 | 67,000 | ,228 |
|  | Roy's Largest Root | ,066 | 1,478^b^ | 3,000 | 67,000 | ,228 |
| typeofexpression * AgexGroup | Pillai's Trace | ,145 | 1,166 | 9,000 | 207,000 | ,318 |
|  | Wilks' Lambda | ,861 | 1,152 | 9,000 | 163,211 | ,329 |
|  | Hotelling's Trace | ,155 | 1,133 | 9,000 | 197,000 | ,341 |
|  | Roy's Largest Root | ,096 | 2,207^c^ | 3,000 | 69,000 | ,095 |
| mod * typeofexpression | Pillai's Trace | ,107 | 2,663^b^ | 3,000 | 67,000 | ,055 |
|  | Wilks' Lambda | ,893 | 2,663^b^ | 3,000 | 67,000 | ,055 |
|  | Hotelling's Trace | ,119 | 2,663^b^ | 3,000 | 67,000 | ,055 |
|  | Roy's Largest Root | ,119 | 2,663^b^ | 3,000 | 67,000 | ,055 |
| mod * typeofexpression * CEGscorebyitem_mean | Pillai's Trace | ,107 | 2,675^b^ | 3,000 | 67,000 | ,054 |
|  | Wilks' Lambda | ,893 | 2,675^b^ | 3,000 | 67,000 | ,054 |
|  | Hotelling's Trace | ,120 | 2,675^b^ | 3,000 | 67,000 | ,054 |
|  | Roy's Largest Root | ,120 | 2,675^b^ | 3,000 | 67,000 | ,054 |
| mod * typeofexpression * AgexGroup | Pillai's Trace | ,155 | 1,249 | 9,000 | 207,000 | ,267 |
|  | Wilks' Lambda | ,850 | 1,248 | 9,000 | 163,211 | ,269 |
|  | Hotelling's Trace | ,170 | 1,241 | 9,000 | 197,000 | ,272 |
|  | Roy's Largest Root | ,126 | 2,908^c^ | 3,000 | 69,000 | ,041 |

| **Tests of Within-Subjects Effects** | | | | | | | |
| --- | --- | --- | --- | --- | --- | --- | --- |
|  | | | | | | | |
| Source | | Type III Sum of Squares | df | Mean Square | F | Sig. | Partial Eta Squared |
| mod | Sphericity Assumed | ,019 | 1 | ,019 | ,771 | ,383 | ,011 |
|  | Greenhouse-Geisser | ,019 | 1,000 | ,019 | ,771 | ,383 | ,011 |
|  | Huynh-Feldt | ,019 | 1,000 | ,019 | ,771 | ,383 | ,011 |
|  | Lower-bound | ,019 | 1,000 | ,019 | ,771 | ,383 | ,011 |
| mod * CEGscorebyitem_mean | Sphericity Assumed | ,020 | 1 | ,020 | ,794 | ,376 | ,011 |
|  | Greenhouse-Geisser | ,020 | 1,000 | ,020 | ,794 | ,376 | ,011 |
|  | Huynh-Feldt | ,020 | 1,000 | ,020 | ,794 | ,376 | ,011 |
|  | Lower-bound | ,020 | 1,000 | ,020 | ,794 | ,376 | ,011 |
| mod * AgexGroup | Sphericity Assumed | ,019 | 3 | ,006 | ,250 | ,861 | ,011 |
|  | Greenhouse-Geisser | ,019 | 3,000 | ,006 | ,250 | ,861 | ,011 |
|  | Huynh-Feldt | ,019 | 3,000 | ,006 | ,250 | ,861 | ,011 |
|  | Lower-bound | ,019 | 3,000 | ,006 | ,250 | ,861 | ,011 |
| Error(mod) | Sphericity Assumed | 1,705 | 69 | ,025 |  |  |  |
|  | Greenhouse-Geisser | 1,705 | 69,000 | ,025 |  |  |  |
|  | Huynh-Feldt | 1,705 | 69,000 | ,025 |  |  |  |
|  | Lower-bound | 1,705 | 69,000 | ,025 |  |  |  |
| typeofexpression | Sphericity Assumed | ,048 | 3 | ,016 | 1,453 | ,229 | ,021 |
|  | Greenhouse-Geisser | ,048 | 2,730 | ,018 | 1,453 | ,231 | ,021 |
|  | Huynh-Feldt | ,048 | 3,000 | ,016 | 1,453 | ,229 | ,021 |
|  | Lower-bound | ,048 | 1,000 | ,048 | 1,453 | ,232 | ,021 |
| typeofexpression * CEGscorebyitem_mean | Sphericity Assumed | ,039 | 3 | ,013 | 1,188 | ,315 | ,017 |
|  | Greenhouse-Geisser | ,039 | 2,730 | ,014 | 1,188 | ,314 | ,017 |
|  | Huynh-Feldt | ,039 | 3,000 | ,013 | 1,188 | ,315 | ,017 |
|  | Lower-bound | ,039 | 1,000 | ,039 | 1,188 | ,279 | ,017 |
| typeofexpression * AgexGroup | Sphericity Assumed | ,118 | 9 | ,013 | 1,189 | ,303 | ,049 |
|  | Greenhouse-Geisser | ,118 | 8,191 | ,014 | 1,189 | ,307 | ,049 |
|  | Huynh-Feldt | ,118 | 9,000 | ,013 | 1,189 | ,303 | ,049 |
|  | Lower-bound | ,118 | 3,000 | ,039 | 1,189 | ,320 | ,049 |
| Error(typeofexpression) | Sphericity Assumed | 2,279 | 207 | ,011 |  |  |  |
|  | Greenhouse-Geisser | 2,279 | 188,388 | ,012 |  |  |  |
|  | Huynh-Feldt | 2,279 | 207,000 | ,011 |  |  |  |
|  | Lower-bound | 2,279 | 69,000 | ,033 |  |  |  |
| mod * typeofexpression | Sphericity Assumed | ,079 | 3 | ,026 | 1,972 | ,119 | ,028 |
|  | Greenhouse-Geisser | ,079 | 2,779 | ,028 | 1,972 | ,124 | ,028 |
|  | Huynh-Feldt | ,079 | 3,000 | ,026 | 1,972 | ,119 | ,028 |
|  | Lower-bound | ,079 | 1,000 | ,079 | 1,972 | ,165 | ,028 |
| mod * typeofexpression * CEGscorebyitem_mean | Sphericity Assumed | ,079 | 3 | ,026 | 1,985 | ,117 | ,028 |
|  | Greenhouse-Geisser | ,079 | 2,779 | ,029 | 1,985 | ,122 | ,028 |
|  | Huynh-Feldt | ,079 | 3,000 | ,026 | 1,985 | ,117 | ,028 |
|  | Lower-bound | ,079 | 1,000 | ,079 | 1,985 | ,163 | ,028 |
| mod * typeofexpression * AgexGroup | Sphericity Assumed | ,187 | 9 | ,021 | 1,560 | ,129 | ,064 |
|  | Greenhouse-Geisser | ,187 | 8,336 | ,022 | 1,560 | ,136 | ,064 |
|  | Huynh-Feldt | ,187 | 9,000 | ,021 | 1,560 | ,129 | ,064 |
|  | Lower-bound | ,187 | 3,000 | ,062 | 1,560 | ,207 | ,064 |
| Error(mod*typeofexpression) | Sphericity Assumed | 2,763 | 207 | ,013 |  |  |  |
|  | Greenhouse-Geisser | 2,763 | 191,724 | ,014 |  |  |  |
|  | Huynh-Feldt | 2,763 | 207,000 | ,013 |  |  |  |
|  | Lower-bound | 2,763 | 69,000 | ,040 |  |  |  |
