# Supplementary material for: Can You Play with Fire and Not Hurt Yourself? A Comparative Study in Figurative Language Comprehension between Individuals with and without Autism Spectrum Disorder
Source: PLoS One. 2016 Dec 30;11(12):e0168571. doi: 10.1371/journal.pone.0168571 (PMC5201294; doi:10.1371/journal.pone.0168571)
Supplement: S7 Appendix — (DOCX) [file pone.0168571.s007.docx]

**Appendix S7. Observed powers**

*Reaction time*

| **Multivariate Tests** | | | | |
| --- | --- | --- | --- | --- |
| Effect | | Partial Eta Squared | Noncent. Parameter | Observed Power^d^ |
| mod | Pillai's Trace | ,004 | ,262 | ,080 |
|  | Wilks' Lambda | ,004 | ,262 | ,080 |
|  | Hotelling's Trace | ,004 | ,262 | ,080 |
|  | Roy's Largest Root | ,004 | ,262 | ,080 |
| mod * CEGscorebyitem_mean | Pillai's Trace | ,003 | ,192 | ,072 |
|  | Wilks' Lambda | ,003 | ,192 | ,072 |
|  | Hotelling's Trace | ,003 | ,192 | ,072 |
|  | Roy's Largest Root | ,003 | ,192 | ,072 |
| mod * AgexGroup | Pillai's Trace | ,026 | 1,991 | ,183 |
|  | Wilks' Lambda | ,026 | 1,991 | ,183 |
|  | Hotelling's Trace | ,026 | 1,991 | ,183 |
|  | Roy's Largest Root | ,026 | 1,991 | ,183 |
| typeofexpression | Pillai's Trace | ,031 | 2,280 | ,205 |
|  | Wilks' Lambda | ,031 | 2,280 | ,205 |
|  | Hotelling's Trace | ,031 | 2,280 | ,205 |
|  | Roy's Largest Root | ,031 | 2,280 | ,205 |
| typeofexpression * CEGscorebyitem_mean | Pillai's Trace | ,043 | 3,239 | ,280 |
|  | Wilks' Lambda | ,043 | 3,239 | ,280 |
|  | Hotelling's Trace | ,043 | 3,239 | ,280 |
|  | Roy's Largest Root | ,043 | 3,239 | ,280 |
| typeofexpression * AgexGroup | Pillai's Trace | ,057 | 13,482 | ,704 |
|  | Wilks' Lambda | ,058 | 10,855 | ,582 |
|  | Hotelling's Trace | ,059 | 13,367 | ,698 |
|  | Roy's Largest Root | ,109 | 9,078 | ,690 |
| mod * typeofexpression | Pillai's Trace | ,049 | 3,709 | ,318 |
|  | Wilks' Lambda | ,049 | 3,709 | ,318 |
|  | Hotelling's Trace | ,049 | 3,709 | ,318 |
|  | Roy's Largest Root | ,049 | 3,709 | ,318 |
| mod * typeofexpression * CEGscorebyitem_mean | Pillai's Trace | ,051 | 3,853 | ,329 |
|  | Wilks' Lambda | ,051 | 3,853 | ,329 |
|  | Hotelling's Trace | ,051 | 3,853 | ,329 |
|  | Roy's Largest Root | ,051 | 3,853 | ,329 |
| mod * typeofexpression * AgexGroup | Pillai's Trace | ,041 | 9,394 | ,513 |
|  | Wilks' Lambda | ,042 | 7,630 | ,413 |
|  | Hotelling's Trace | ,043 | 9,483 | ,517 |
|  | Roy's Largest Root | ,105 | 8,654 | ,667 |

| **Tests of Within-Subjects Effects** | | | |
| --- | --- | --- | --- |
|  | | | |
| Source | | Noncent. Parameter | Observed Power^a^ |
| mod | Sphericity Assumed | ,262 | ,080 |
|  | Greenhouse-Geisser | ,262 | ,080 |
|  | Huynh-Feldt | ,262 | ,080 |
|  | Lower-bound | ,262 | ,080 |
| mod * CEGscorebyitem_mean | Sphericity Assumed | ,192 | ,072 |
|  | Greenhouse-Geisser | ,192 | ,072 |
|  | Huynh-Feldt | ,192 | ,072 |
|  | Lower-bound | ,192 | ,072 |
| mod * AgexGroup | Sphericity Assumed | 1,991 | ,183 |
|  | Greenhouse-Geisser | 1,991 | ,183 |
|  | Huynh-Feldt | 1,991 | ,183 |
|  | Lower-bound | 1,991 | ,183 |
| Error(mod) | Sphericity Assumed |  |  |
|  | Greenhouse-Geisser |  |  |
|  | Huynh-Feldt |  |  |
|  | Lower-bound |  |  |
| typeofexpression | Sphericity Assumed | ,978 | ,113 |
|  | Greenhouse-Geisser | ,634 | ,101 |
|  | Huynh-Feldt | ,686 | ,103 |
|  | Lower-bound | ,326 | ,087 |
| typeofexpression * CEGscorebyitem_mean | Sphericity Assumed | 1,701 | ,166 |
|  | Greenhouse-Geisser | 1,103 | ,141 |
|  | Huynh-Feldt | 1,194 | ,145 |
|  | Lower-bound | ,567 | ,115 |
| typeofexpression * AgexGroup | Sphericity Assumed | 11,134 | ,601 |
|  | Greenhouse-Geisser | 7,220 | ,468 |
|  | Huynh-Feldt | 7,813 | ,490 |
|  | Lower-bound | 3,711 | ,318 |
| Error(typeofexpression) | Sphericity Assumed |  |  |
|  | Greenhouse-Geisser |  |  |
|  | Huynh-Feldt |  |  |
|  | Lower-bound |  |  |
| mod * typeofexpression | Sphericity Assumed | 2,645 | ,241 |
|  | Greenhouse-Geisser | 1,302 | ,176 |
|  | Huynh-Feldt | 1,394 | ,181 |
|  | Lower-bound | ,882 | ,153 |
| mod * typeofexpression * CEGscorebyitem_mean | Sphericity Assumed | 2,930 | ,265 |
|  | Greenhouse-Geisser | 1,443 | ,191 |
|  | Huynh-Feldt | 1,545 | ,196 |
|  | Lower-bound | ,977 | ,164 |
| mod * typeofexpression * AgexGroup | Sphericity Assumed | 5,954 | ,323 |
|  | Greenhouse-Geisser | 2,932 | ,220 |
|  | Huynh-Feldt | 3,139 | ,227 |
|  | Lower-bound | 1,985 | ,183 |
| Error(mod*typeofexpression) | Sphericity Assumed |  |  |
|  | Greenhouse-Geisser |  |  |
|  | Huynh-Feldt |  |  |
|  | Lower-bound |  |  |

*Overall accuracy*

| **Multivariate Tests** | | | | |
| --- | --- | --- | --- | --- |
| Effect | | Partial Eta Squared | Noncent. Parameter | Observed Power^d^ |
| mod | Pillai's Trace | ,007 | ,538 | ,112 |
|  | Wilks' Lambda | ,007 | ,538 | ,112 |
|  | Hotelling's Trace | ,007 | ,538 | ,112 |
|  | Roy's Largest Root | ,007 | ,538 | ,112 |
| mod * CEGscorebyitem_mean | Pillai's Trace | ,008 | ,586 | ,117 |
|  | Wilks' Lambda | ,008 | ,586 | ,117 |
|  | Hotelling's Trace | ,008 | ,586 | ,117 |
|  | Roy's Largest Root | ,008 | ,586 | ,117 |
| mod * AgexGroup | Pillai's Trace | ,039 | 2,983 | ,260 |
|  | Wilks' Lambda | ,039 | 2,983 | ,260 |
|  | Hotelling's Trace | ,039 | 2,983 | ,260 |
|  | Roy's Largest Root | ,039 | 2,983 | ,260 |
| typeofexpression | Pillai's Trace | ,016 | 1,146 | ,122 |
|  | Wilks' Lambda | ,016 | 1,146 | ,122 |
|  | Hotelling's Trace | ,016 | 1,146 | ,122 |
|  | Roy's Largest Root | ,016 | 1,146 | ,122 |
| typeofexpression * CEGscorebyitem_mean | Pillai's Trace | ,013 | ,950 | ,109 |
|  | Wilks' Lambda | ,013 | ,950 | ,109 |
|  | Hotelling's Trace | ,013 | ,950 | ,109 |
|  | Roy's Largest Root | ,013 | ,950 | ,109 |
| typeofexpression * AgexGroup | Pillai's Trace | ,027 | 6,049 | ,329 |
|  | Wilks' Lambda | ,027 | 4,821 | ,258 |
|  | Hotelling's Trace | ,027 | 5,879 | ,319 |
|  | Roy's Largest Root | ,053 | 4,142 | ,353 |
| mod * typeofexpression | Pillai's Trace | ,005 | ,352 | ,070 |
|  | Wilks' Lambda | ,005 | ,352 | ,070 |
|  | Hotelling's Trace | ,005 | ,352 | ,070 |
|  | Roy's Largest Root | ,005 | ,352 | ,070 |
| mod * typeofexpression * CEGscorebyitem_mean | Pillai's Trace | ,004 | ,254 | ,065 |
|  | Wilks' Lambda | ,004 | ,254 | ,065 |
|  | Hotelling's Trace | ,004 | ,254 | ,065 |
|  | Roy's Largest Root | ,004 | ,254 | ,065 |
| mod * typeofexpression * AgexGroup | Pillai's Trace | ,045 | 10,551 | ,572 |
|  | Wilks' Lambda | ,047 | 8,585 | ,465 |
|  | Hotelling's Trace | ,048 | 10,687 | ,578 |
|  | Roy's Largest Root | ,114 | 9,537 | ,714 |

| **Tests of Within-Subjects Effects** | | | |
| --- | --- | --- | --- |
|  | | | |
| Source | | Noncent. Parameter | Observed Power^a^ |
| mod | Sphericity Assumed | ,538 | ,112 |
|  | Greenhouse-Geisser | ,538 | ,112 |
|  | Huynh-Feldt | ,538 | ,112 |
|  | Lower-bound | ,538 | ,112 |
| mod * CEGscorebyitem_mean | Sphericity Assumed | ,586 | ,117 |
|  | Greenhouse-Geisser | ,586 | ,117 |
|  | Huynh-Feldt | ,586 | ,117 |
|  | Lower-bound | ,586 | ,117 |
| mod * AgexGroup | Sphericity Assumed | 2,983 | ,260 |
|  | Greenhouse-Geisser | 2,983 | ,260 |
|  | Huynh-Feldt | 2,983 | ,260 |
|  | Lower-bound | 2,983 | ,260 |
| Error(mod) | Sphericity Assumed |  |  |
|  | Greenhouse-Geisser |  |  |
|  | Huynh-Feldt |  |  |
|  | Lower-bound |  |  |
| typeofexpression | Sphericity Assumed | ,884 | ,106 |
|  | Greenhouse-Geisser | ,685 | ,100 |
|  | Huynh-Feldt | ,747 | ,102 |
|  | Lower-bound | ,295 | ,083 |
| typeofexpression * CEGscorebyitem_mean | Sphericity Assumed | ,900 | ,107 |
|  | Greenhouse-Geisser | ,698 | ,101 |
|  | Huynh-Feldt | ,761 | ,103 |
|  | Lower-bound | ,300 | ,084 |
| typeofexpression * AgexGroup | Sphericity Assumed | 5,177 | ,280 |
|  | Greenhouse-Geisser | 4,013 | ,244 |
|  | Huynh-Feldt | 4,375 | ,255 |
|  | Lower-bound | 1,726 | ,164 |
| Error(typeofexpression) | Sphericity Assumed |  |  |
|  | Greenhouse-Geisser |  |  |
|  | Huynh-Feldt |  |  |
|  | Lower-bound |  |  |
| mod * typeofexpression | Sphericity Assumed | ,399 | ,074 |
|  | Greenhouse-Geisser | ,341 | ,072 |
|  | Huynh-Feldt | ,373 | ,073 |
|  | Lower-bound | ,133 | ,065 |
| mod * typeofexpression * CEGscorebyitem_mean | Sphericity Assumed | ,277 | ,066 |
|  | Greenhouse-Geisser | ,236 | ,065 |
|  | Huynh-Feldt | ,259 | ,066 |
|  | Lower-bound | ,092 | ,060 |
| mod * typeofexpression * AgexGroup | Sphericity Assumed | 14,697 | ,749 |
|  | Greenhouse-Geisser | 12,570 | ,696 |
|  | Huynh-Feldt | 13,764 | ,727 |
|  | Lower-bound | 4,899 | ,412 |
| Error(mod*typeofexpression) | Sphericity Assumed |  |  |
|  | Greenhouse-Geisser |  |  |
|  | Huynh-Feldt |  |  |
|  | Lower-bound |  |  |

*Comparison between target and literal responses*

| **Multivariate Tests** | | | | |
| --- | --- | --- | --- | --- |
| Effect | | Partial Eta Squared | Noncent. Parameter | Observed Power^d^ |
| mod | Pillai's Trace | ,013 | ,956 | ,162 |
|  | Wilks' Lambda | ,013 | ,956 | ,162 |
|  | Hotelling's Trace | ,013 | ,956 | ,162 |
|  | Roy's Largest Root | ,013 | ,956 | ,162 |
| mod * CEGscorebyitem_mean | Pillai's Trace | ,013 | ,953 | ,161 |
|  | Wilks' Lambda | ,013 | ,953 | ,161 |
|  | Hotelling's Trace | ,013 | ,953 | ,161 |
|  | Roy's Largest Root | ,013 | ,953 | ,161 |
| mod * AgexGroup | Pillai's Trace | ,021 | 1,556 | ,151 |
|  | Wilks' Lambda | ,021 | 1,556 | ,151 |
|  | Hotelling's Trace | ,021 | 1,556 | ,151 |
|  | Roy's Largest Root | ,021 | 1,556 | ,151 |
| typeofexpression | Pillai's Trace | ,031 | 2,341 | ,210 |
|  | Wilks' Lambda | ,031 | 2,341 | ,210 |
|  | Hotelling's Trace | ,031 | 2,341 | ,210 |
|  | Roy's Largest Root | ,031 | 2,341 | ,210 |
| typeofexpression * CEGscorebyitem_mean | Pillai's Trace | ,025 | 1,851 | ,173 |
|  | Wilks' Lambda | ,025 | 1,851 | ,173 |
|  | Hotelling's Trace | ,025 | 1,851 | ,173 |
|  | Roy's Largest Root | ,025 | 1,851 | ,173 |
| typeofexpression * AgexGroup | Pillai's Trace | ,016 | 3,578 | ,196 |
|  | Wilks' Lambda | ,016 | 2,834 | ,158 |
|  | Hotelling's Trace | ,016 | 3,435 | ,188 |
|  | Roy's Largest Root | ,029 | 2,176 | ,197 |
| mod * typeofexpression | Pillai's Trace | ,008 | ,571 | ,084 |
|  | Wilks' Lambda | ,008 | ,571 | ,084 |
|  | Hotelling's Trace | ,008 | ,571 | ,084 |
|  | Roy's Largest Root | ,008 | ,571 | ,084 |
| mod * typeofexpression * CEGscorebyitem_mean | Pillai's Trace | ,007 | ,483 | ,078 |
|  | Wilks' Lambda | ,007 | ,483 | ,078 |
|  | Hotelling's Trace | ,007 | ,483 | ,078 |
|  | Roy's Largest Root | ,007 | ,483 | ,078 |
| mod * typeofexpression * AgexGroup | Pillai's Trace | ,049 | 11,523 | ,619 |
|  | Wilks' Lambda | ,050 | 9,217 | ,499 |
|  | Hotelling's Trace | ,051 | 11,281 | ,607 |
|  | Roy's Largest Root | ,095 | 7,813 | ,617 |

| **Tests of Within-Subjects Effects** | | | |
| --- | --- | --- | --- |
|  | | | |
| Source | | Noncent. Parameter | Observed Power^a^ |
| mod | Sphericity Assumed | ,956 | ,162 |
|  | Greenhouse-Geisser | ,956 | ,162 |
|  | Huynh-Feldt | ,956 | ,162 |
|  | Lower-bound | ,956 | ,162 |
| mod * CEGscorebyitem_mean | Sphericity Assumed | ,953 | ,161 |
|  | Greenhouse-Geisser | ,953 | ,161 |
|  | Huynh-Feldt | ,953 | ,161 |
|  | Lower-bound | ,953 | ,161 |
| mod * AgexGroup | Sphericity Assumed | 1,556 | ,151 |
|  | Greenhouse-Geisser | 1,556 | ,151 |
|  | Huynh-Feldt | 1,556 | ,151 |
|  | Lower-bound | 1,556 | ,151 |
| Error(mod) | Sphericity Assumed |  |  |
|  | Greenhouse-Geisser |  |  |
|  | Huynh-Feldt |  |  |
|  | Lower-bound |  |  |
| typeofexpression | Sphericity Assumed | 3,200 | ,287 |
|  | Greenhouse-Geisser | 2,378 | ,247 |
|  | Huynh-Feldt | 2,588 | ,257 |
|  | Lower-bound | 1,067 | ,175 |
| typeofexpression * CEGscorebyitem_mean | Sphericity Assumed | 2,742 | ,249 |
|  | Greenhouse-Geisser | 2,038 | ,216 |
|  | Huynh-Feldt | 2,218 | ,225 |
|  | Lower-bound | ,914 | ,157 |
| typeofexpression * AgexGroup | Sphericity Assumed | 3,544 | ,194 |
|  | Greenhouse-Geisser | 2,634 | ,169 |
|  | Huynh-Feldt | 2,867 | ,176 |
|  | Lower-bound | 1,181 | ,124 |
| Error(typeofexpression) | Sphericity Assumed |  |  |
|  | Greenhouse-Geisser |  |  |
|  | Huynh-Feldt |  |  |
|  | Lower-bound |  |  |
| mod * typeofexpression | Sphericity Assumed | ,843 | ,103 |
|  | Greenhouse-Geisser | ,634 | ,096 |
|  | Huynh-Feldt | ,691 | ,098 |
|  | Lower-bound | ,281 | ,082 |
| mod * typeofexpression * CEGscorebyitem_mean | Sphericity Assumed | ,703 | ,094 |
|  | Greenhouse-Geisser | ,529 | ,088 |
|  | Huynh-Feldt | ,577 | ,090 |
|  | Lower-bound | ,234 | ,077 |
| mod * typeofexpression * AgexGroup | Sphericity Assumed | 13,554 | ,707 |
|  | Greenhouse-Geisser | 10,204 | ,610 |
|  | Huynh-Feldt | 11,110 | ,638 |
|  | Lower-bound | 4,518 | ,382 |
| Error(mod*typeofexpression) | Sphericity Assumed |  |  |
|  | Greenhouse-Geisser |  |  |
|  | Huynh-Feldt |  |  |
|  | Lower-bound |  |  |

*Comparison between target and figurative non target responses*

| **Multivariate Tests** | | | | |
| --- | --- | --- | --- | --- |
| Effect | | Partial Eta Squared | Noncent. Parameter | Observed Power^d^ |
| mod | Pillai's Trace | ,011 | ,771 | ,139 |
|  | Wilks' Lambda | ,011 | ,771 | ,139 |
|  | Hotelling's Trace | ,011 | ,771 | ,139 |
|  | Roy's Largest Root | ,011 | ,771 | ,139 |
| mod * CEGscorebyitem_mean | Pillai's Trace | ,011 | ,794 | ,142 |
|  | Wilks' Lambda | ,011 | ,794 | ,142 |
|  | Hotelling's Trace | ,011 | ,794 | ,142 |
|  | Roy's Largest Root | ,011 | ,794 | ,142 |
| mod * AgexGroup | Pillai's Trace | ,011 | ,750 | ,095 |
|  | Wilks' Lambda | ,011 | ,750 | ,095 |
|  | Hotelling's Trace | ,011 | ,750 | ,095 |
|  | Roy's Largest Root | ,011 | ,750 | ,095 |
| typeofexpression | Pillai's Trace | ,073 | 5,251 | ,437 |
|  | Wilks' Lambda | ,073 | 5,251 | ,437 |
|  | Hotelling's Trace | ,073 | 5,251 | ,437 |
|  | Roy's Largest Root | ,073 | 5,251 | ,437 |
| typeofexpression * CEGscorebyitem_mean | Pillai's Trace | ,062 | 4,435 | ,374 |
|  | Wilks' Lambda | ,062 | 4,435 | ,374 |
|  | Hotelling's Trace | ,062 | 4,435 | ,374 |
|  | Roy's Largest Root | ,062 | 4,435 | ,374 |
| typeofexpression * AgexGroup | Pillai's Trace | ,048 | 10,495 | ,568 |
|  | Wilks' Lambda | ,049 | 8,363 | ,452 |
|  | Hotelling's Trace | ,049 | 10,200 | ,552 |
|  | Roy's Largest Root | ,088 | 6,620 | ,537 |
| mod * typeofexpression | Pillai's Trace | ,107 | 7,990 | ,625 |
|  | Wilks' Lambda | ,107 | 7,990 | ,625 |
|  | Hotelling's Trace | ,107 | 7,990 | ,625 |
|  | Roy's Largest Root | ,107 | 7,990 | ,625 |
| mod * typeofexpression * CEGscorebyitem_mean | Pillai's Trace | ,107 | 8,025 | ,627 |
|  | Wilks' Lambda | ,107 | 8,025 | ,627 |
|  | Hotelling's Trace | ,107 | 8,025 | ,627 |
|  | Roy's Largest Root | ,107 | 8,025 | ,627 |
| mod * typeofexpression * AgexGroup | Pillai's Trace | ,052 | 11,243 | ,604 |
|  | Wilks' Lambda | ,053 | 9,058 | ,489 |
|  | Hotelling's Trace | ,054 | 11,170 | ,600 |
|  | Roy's Largest Root | ,112 | 8,724 | ,669 |

| **Tests of Within-Subjects Effects** | | | |
| --- | --- | --- | --- |
|  | | | |
| Source | | Noncent. Parameter | Observed Power^a^ |
| mod | Sphericity Assumed | ,771 | ,139 |
|  | Greenhouse-Geisser | ,771 | ,139 |
|  | Huynh-Feldt | ,771 | ,139 |
|  | Lower-bound | ,771 | ,139 |
| mod * CEGscorebyitem_mean | Sphericity Assumed | ,794 | ,142 |
|  | Greenhouse-Geisser | ,794 | ,142 |
|  | Huynh-Feldt | ,794 | ,142 |
|  | Lower-bound | ,794 | ,142 |
| mod * AgexGroup | Sphericity Assumed | ,750 | ,095 |
|  | Greenhouse-Geisser | ,750 | ,095 |
|  | Huynh-Feldt | ,750 | ,095 |
|  | Lower-bound | ,750 | ,095 |
| Error(mod) | Sphericity Assumed |  |  |
|  | Greenhouse-Geisser |  |  |
|  | Huynh-Feldt |  |  |
|  | Lower-bound |  |  |
| typeofexpression | Sphericity Assumed | 4,358 | ,382 |
|  | Greenhouse-Geisser | 3,966 | ,362 |
|  | Huynh-Feldt | 4,358 | ,382 |
|  | Lower-bound | 1,453 | ,221 |
| typeofexpression * CEGscorebyitem_mean | Sphericity Assumed | 3,565 | ,317 |
|  | Greenhouse-Geisser | 3,245 | ,301 |
|  | Huynh-Feldt | 3,565 | ,317 |
|  | Lower-bound | 1,188 | ,189 |
| typeofexpression * AgexGroup | Sphericity Assumed | 10,704 | ,578 |
|  | Greenhouse-Geisser | 9,741 | ,548 |
|  | Huynh-Feldt | 10,704 | ,578 |
|  | Lower-bound | 3,568 | ,306 |
| Error(typeofexpression) | Sphericity Assumed |  |  |
|  | Greenhouse-Geisser |  |  |
|  | Huynh-Feldt |  |  |
|  | Lower-bound |  |  |
| mod * typeofexpression | Sphericity Assumed | 5,917 | ,504 |
|  | Greenhouse-Geisser | 5,481 | ,483 |
|  | Huynh-Feldt | 5,917 | ,504 |
|  | Lower-bound | 1,972 | ,283 |
| mod * typeofexpression * CEGscorebyitem_mean | Sphericity Assumed | 5,955 | ,506 |
|  | Greenhouse-Geisser | 5,515 | ,485 |
|  | Huynh-Feldt | 5,955 | ,506 |
|  | Lower-bound | 1,985 | ,285 |
| mod * typeofexpression * AgexGroup | Sphericity Assumed | 14,039 | ,724 |
|  | Greenhouse-Geisser | 13,003 | ,698 |
|  | Huynh-Feldt | 14,039 | ,724 |
|  | Lower-bound | 4,680 | ,394 |
| Error(mod*typeofexpression) | Sphericity Assumed |  |  |
|  | Greenhouse-Geisser |  |  |
|  | Huynh-Feldt |  |  |
|  | Lower-bound |  |  |
